# Effect of a Peer Health Coaching Intervention on Clinical Outcomes Among US Veterans With Cardiovascular Risks: The Vet-COACH Randomized Clinical Trial

**DOI:** 10.1001/jamanetworkopen.2023.17046

**Published:** 2023-06-06

**Authors:** Karin M. Nelson, Leslie Taylor, Jennifer L. Williams, Mayuree Rao, Kristen E. Gray, Charles Bradley Kramer, Eric Epler, Tiffanie Fennell

**Affiliations:** 1 Health Services Research and Development Center of Innovation, Veterans Affairs (VA) Puget Sound Health Care System, Seattle, Washington; 2 General Internal Medicine Service, VA Puget Sound Health Care System, Seattle, Washington; 3 Department of Medicine, University of Washington School of Medicine, Seattle; 4 Department of Health Systems and Population Health, University of Washington School of Public Health, Seattle; 5 Department of Psychiatry and Behavioral Sciences, University of Washington School of Medicine, Seattle

## Abstract

**Importance:**

Although cardiovascular disease (CVD) is the leading cause of death in the US, CVD risk factors remain suboptimally controlled.

**Objective:**

To test the effectiveness of a home-visit, peer health coaching intervention to improve health outcomes for veterans with multiple CVD risks.

**Design, Setting, and Participants:**

This 2-group, unblinded randomized clinical trial, called Vet-COACH (Veteran Peer Coaches Optimizing and Advancing Cardiac Health), used a novel geographic-based method to recruit a racially diverse population of veterans with low income. These veterans were enrolled at the Seattle or American Lake Veterans Health Affairs primary care clinics in Washington state. Veterans with a diagnosis of hypertension with at least 1 blood pressure reading of 150/90 mm Hg or higher in the past year, and 1 other CVD risk factor (current smoker, overweight or obesity, and/or hyperlipidemia), who resided in Census tracts with the highest prevalence of hypertension were eligible to participate. Participants were randomized to the intervention group (n = 134) or control group (n = 130). An intention-to-treat analysis was performed from May 2017 to October 2021.

**Intervention:**

Participants in the intervention group received peer health coaching for 12 months with mandatory and optional educational materials, an automatic blood pressure monitor, a scale, a pill organizer, and healthy nutrition tools. Participants in the control group received usual care plus educational materials.

**Main Outcomes and Measures:**

The primary outcome was a change in systolic blood pressure (SBP) from baseline to 12-month follow-up. Secondary outcomes included change in health-related quality of life (HRQOL; measured using the 12-item Short Form survey’s Mental Component Summary and Physical Component Summary scores), Framingham Risk Score, and overall CVD risk and health care use (hospitalizations, emergency department visits, and outpatient visits).

**Results:**

The 264 participants who were randomized (mean [SD] age of 60.6 [9.7] years) were predominantly male (229 [87%]) and 73 (28%) were Black individuals and 103 (44%) reported low annual income (<$40 000 per year). Seven peer health coaches were recruited. No difference was found in change in SBP between the intervention and control groups (−3.32 [95% CI, −6.88 to 0.23] mm Hg vs −0.40 [95% CI, −4.20 to 3.39] mm Hg; adjusted difference in differences, −2.05 [95% CI, −7.00 to 2.55] mm Hg; *P* = .40). Participants in the intervention vs control group reported greater improvements in mental HRQOL scores (2.19 [95% CI, 0.26-4.12] points vs −1.01 [95% CI, −2.91 to 0.88] points; adjusted difference in differences, 3.64 [95% CI, 0.66-6.63] points; *P* = .02). No difference was found in physical HRQOL scores, Framingham Risk Scores, and overall CVD risk or health care use.

**Conclusions and Relevance:**

This trial found that, although the peer health coaching program did not significantly decrease SBP, participants who received the intervention reported better mental HRQOL compared with the control group. The results suggest that a peer-support model that is integrated into primary care can create opportunities for well-being improvements beyond blood pressure control.

**Trial Registration:**

ClinicalTrials.gov Identifier: NCT02697422

## Key Points

**Question:**

Is a home visit from a peer health coach effective at improving systolic blood pressure (SBP) and other clinical outcomes for veterans with multiple cardiovascular disease (CVD) risks?

**Findings:**

In this randomized clinical trial of 264 US veterans, there was no significant improvement in SBP in participants who received the peer health coaching intervention. However, a significant improvement in the mental health–related quality of life was reported in the intervention group compared with the control group.

**Meaning:**

Findings from this trial indicate that a peer health coaching intervention can create opportunities for well-being improvements beyond blood pressure control.

## Teaser

This randomized clinical trial assesses changes in systolic blood pressure, health-related quality of life, cardiovascular risk, and health care use associated with use of peer health coaching among military veterans who use the Veterans Health Affairs system.

## Introduction

Cardiovascular disease (CVD) is the leading cause of death in the US, and non-Hispanic Black Americans have a higher burden of CVD than other groups.^1,2,3^ Cardiovascular risk factors remain suboptimally controlled in the US population^4^ and among veterans who use the Veterans Health Administration (VHA) for care.^5,6,7^ Almost one-half of these veterans have a diagnosis of hypertension, and one-quarter have poor blood pressure control.^5^ Veterans who obtain care from VHA are more likely to have obesity and be physically inactive than the general population, and 25% use tobacco.^7^ These characteristics make veterans a group for whom CVD risk reduction is especially warranted.

Successful management of cardiovascular risks is a complex process that requires self-monitoring, using medications, adjusting diet and physical activity, and working effectively with health care clinicians.^8^ Various patient and clinician-facing interventions to improve cardiovascular risk have been trialed in the VHA with mixed results.^9,10,11^ Peer support has been shown to improve health outcomes for patients with chronic conditions^12,13,14,15^; however, there is less evidence for chronic disease prevention and how to integrate peer support into a team-based primary care clinic.

The goal of this randomized clinical trial, Veteran Peer Coaches Optimizing and Advancing Cardiac Health (Vet-COACH), was to test the effectiveness of a home-visit, peer health coaching intervention to improve health outcomes for veterans with multiple CVD risks. We used a neighborhood-based recruitment strategy for both the coaches and participants, targeting areas with a disproportionate number of people with cardiovascular risks.

## Methods

### Study Design, Setting, and Participants

A description of the trial design, intervention components, and peer health coach training has been previously published.^16^ We conducted the Vet-COACH trial from May 2017 to October 2021 involving veterans with hypertension and 1 other CVD risk factor. The Veterans Affairs Puget Sound Health Care System Institutional Review Board approved the trial protocol (Supplement 1). All participants provided written informed consent. We followed the Consolidated Standards of Reporting Trials (CONSORT) reporting guideline.

Veterans were eligible to participate if they had a diagnosis of hypertension (with an *International Statistical Classification of Diseases, Tenth Revision, Clinical Modification* code 401 [essential hypertension]), at least 1 blood pressure measurement of 150/90 mm Hg or higher that was documented in the medical record in the preceding 12 months, and 1 other self-reported CVD risk (current smoking, overweight or obesity, and/or diagnosis of hyperlipidemia) on the initial telephone screening. Eligible participants were enrolled at the Seattle or American Lake VHA primary care and women’s health clinics in Washington state. We recruited both peer health coaches and participants who lived in Census tracts with the highest rates of hypertension among the clinic population.^16^

We sent recruitment letters to 2921 potentially eligible veterans. Of the 1419 veterans we were able to contact, 425 agreed to complete an additional screening to determine eligibility, of whom 264 were randomized (Figure). The trial was stopped prior to achieving recruitment goals due to budget and staffing constraints, in addition to the COVID-19 pandemic. The CONSORT diagram shows the participant flow (Figure).

**Figure.**
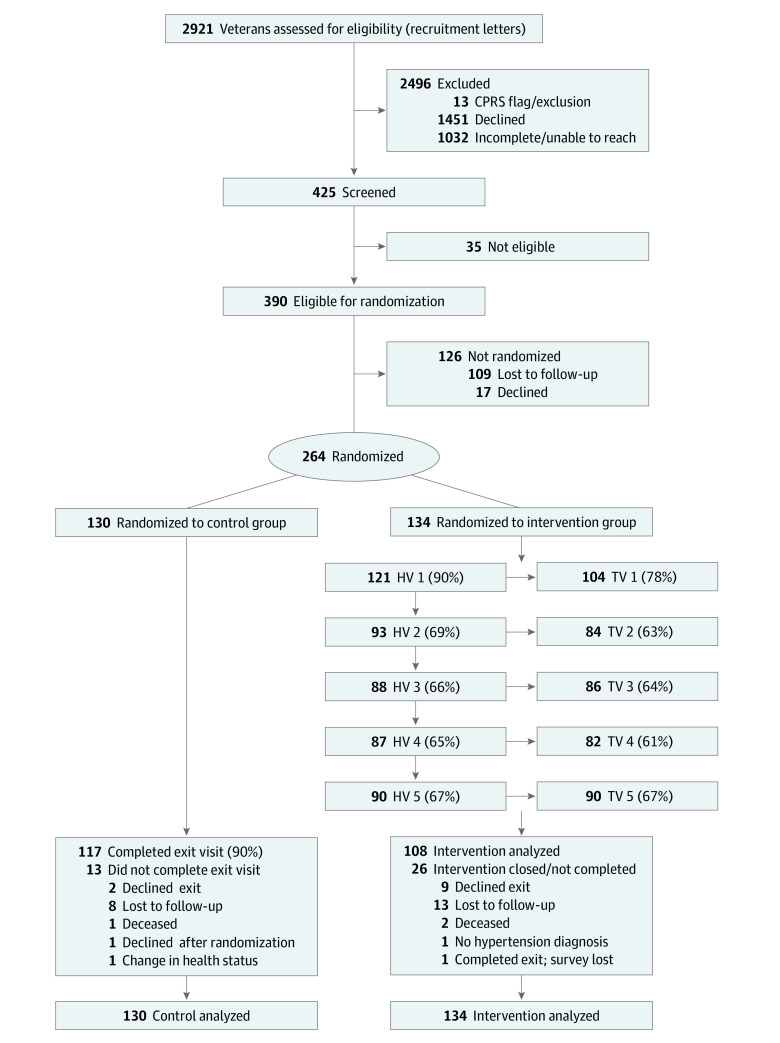
Participant Flow Diagram CPRS indicates Computerized Patient Record System; HV, home visit; TV, telephone visit.

After completing baseline data collection, participants were randomized to receive either a home-based peer-support intervention for 12 months or usual care. Block randomization with varying blocks of 4 or 6 was used to ensure the treatment groups were not imbalanced at any time. Concealment was used to prevent study staff from obtaining information on the sequence of assignment during the baseline assessment. The trial was unblinded; the same staff conducted both the research visits and peer health coach supervision.

### Vet-COACH Intervention

Of the 7 peer health coaches, 1 was assigned to a participant in each intervention arm and made 5 home visits and 5 telephone visits over 12 months. Telephone visits were used between in-home visits as check-ins on goal setting and progress. The protocol was modified to telephone-only visits after the start of the COVID-19 pandemic in March 2020.

Peer health coaches assessed blood pressure control, provided social support, targeted health education, assisted participants with health goal setting, and linked participants to clinic and community-based resources. Five required modules were completed during health coaching sessions: blood pressure, physical activity, nutrition, medication adherence, and communication with medical team or physician. Participants could review nonmandatory modules, such as weight management, smoking cessation, limiting alcohol intake, and managing stress, according to their personal interests and needs. Participants received a manual of educational materials, an automatic blood pressure monitor, a scale, a pill organizer, and healthy nutrition tools.^17^

To monitor intervention fidelity, peer health coaches completed an encounter form at each visit to document the educational content, progress toward previously set goals, and referrals. Ongoing assessments were used to ensure that the content delivered by the peer health coach adhered to 80% or more of the intervention components. Each peer health coach audio recorded the first home visit for each participant until the study team ensured fidelity. A 5% random sample of visits were audio recorded and transcribed for continued fidelity assessment and peer health coach training.

The intervention was linked to VHA primary care in several ways. All participants were required to be enrolled in and active users of VHA primary care. We used VHA criteria to define study eligibility criteria for veterans with out-of-range blood pressure levels. Peer health coaches communicated with the study team regarding urgent issues in addition to attending meetings every other week to present cases. The peer health coaches encouraged participants to follow-up with their primary care clinic for routine care and when their blood pressure was elevated beyond the normal range. The study team communicated elevated blood pressure readings and other clinical needs (eg, appointment requests, medication refills, or other referrals) to primary care clinical staff via the electronic health record or direct contact.

Participants in the control group received usual medical care plus the same educational materials provided to the intervention group. Elevated blood pressure readings at study enrollment and exit visits were reported to their primary care team.

### Outcomes and Measures

The primary outcome was a change in systolic blood pressure (SBP) from baseline to the 12-month follow-up. Blood pressure readings were obtained using standard procedures with a blood pressure monitor (UA-767F Multi-User Blood Pressure Monitor; A&D Medical). The mean of 3 blood pressure measurements was used. If a participant did not complete an exit visit, blood pressure readings were extracted from the medical record for a 3-month period around the date of the missed exit visit (using the lowest value for the selected day at an outpatient visit, excluding inpatient and emergency department [ED] values). Mean blood pressure readings were categorized using standard criteria.^18^

A dichotomous secondary outcome was defined as the percentage of respondents meeting prespecified normal blood pressure goal (≤120/80 mm Hg).^18^ During the COVID-19 pandemic, all study procedures were conducted by telephone, including measurement of blood pressure. Participants were instructed by trained research staff to use their home blood pressure monitor to take and self-report their blood pressure readings. Additional secondary outcomes included diastolic blood pressure, health-related quality of life (HRQOL), cardiovascular risk, and prior year health care use, measured at baseline and 12 months. The HRQOL was measured with the 12-item Short Form (SF-12) survey,^19,20^ which reflects general health status and leads to 2 scores: Physical Component Summary (PCS) and Mental Component Summary (MCS). The MCS and PCS scores range from 0 to 100 points (the mean is scaled to be 50 points for the US population), with lower scores indicating poorer mental and physical HRQOL, respectively. A nonfasting lipid panel (total cholesterol, low-density lipoprotein cholesterol, high-density lipoprotein cholesterol, and triglycerides) was used to calculate the Framingham Risk Score,^21^ a 10-year estimate of CVD risk ranging from 0% to greater than 30%. Body mass index (BMI) was calculated as weight in kilograms divided by height in meters squared. Self-reported smoking status was obtained using validated questions.^22^ We collected data on prior year health care use (hospitalizations, ED use, and outpatient primary care encounters) from VHA administrative data.

Self-reported demographic characteristics included age, sex, marital status, educational level, annual income, and race and ethnicity. Race and ethnicity were studied due to the distribution of CVD risk factors in the US. Participants were asked to identify their race from 1 or more of the following categories: American Indian or Alaska Native, Asian and Pacific Islander, Black, White, or multiracial.^22^ Participants were asked to define their ethnicity as Hispanic, Latino, or Spanish origin or as non-Hispanic.

### Statistical Analysis

We projected a sample size of 400 veterans to detect a clinically significant difference between intervention and control groups in SBP change of 5 mm Hg between baseline and 12-month follow-up.^23,24^ We calculated a power of .80 and an α = .05, accounted for 15% loss to follow-up, and clustered by peer health coach and primary care clinician.^25,26^

We examined distributions of demographic and clinical variables of participants in the intervention and control arms and reported summary statistics for all baseline covariates and outcomes (means [SDs] for continuous variables; frequencies [percents] for categorical variables) by randomized group. We examined the association between baseline measures and outcome variables by randomized group for the linearity and equality of slopes necessary for use in subsequent statistical models. All analyses were performed with R, version 4.3.0 (R Foundation for Statistical Computing), and SAS, version 9.4 (SAS Institute Inc).

Primary intent-to-treat analyses compared changes in SBP from baseline to 12 months between the intervention and control groups using a mixed-effects model adjusted for baseline SBP values and treatment group. We included a physician-level and peer health coach–level (for intervention group only) random effect to account for the correlation of multiple patients per physician with multiple patients per peer health coach.^27^ We compared baseline and 12-month differences in continuous secondary outcomes (except for health care use) between treatment groups using the same model. For binary outcomes, we modeled 12-month values adjusted for the same variables. For the health care use outcomes, we modeled 12-month outcomes (number of hospital admissions, ED visits, and primary care outpatient visits) using a 2-stage model approach to account for the excessive 0 counts: a logistic regression model for the binary positive-count indicator and a γ or Poisson model for positive counts adjusted for baseline values, intervention group, and random physician and peer health coach effects. We reported adjusted differences in 12-month use between treatment groups with bootstrapped CIs.^28^ All hypotheses were tested with a 2-sided *P* = .05.

We performed prespecified sensitivity analyses to examine whether number of coach visits influenced the effect of the intervention on the primary outcome. We also conducted a post hoc stratified analysis before and after the start of the COVID-19 pandemic since trial activities were conducted remotely after March 2020. Participants with 12-month outcomes that were captured before March 5, 2020, were defined as the pre–COVID-19 subgroup, and the remaining participants were defined as the post–COVID-19 subgroup. The post–COVID-19 subgroup had 128 participants (64 control participants; 64 intervention participants). Because the SF-12 had 15% missing values, we analyzed patient and clinical factors that were associated with missingness. We performed multiple imputation using mice package in R to impute results to allow analysis for all participants and to reduce any bias associated with the missing data (eMethods and eTables 1 and 2 in Supplement 2).^29^ Additional information on the study design and statistical analysis plan is provided in Supplement 1.

## Results

### Trial Participants

Of the 264 randomized participants (130 to control group; 134 to intervention group), 225 completed the 12-month follow-up (85% completion rate). Eighty participants (60%) in the intervention group received at least 8 of the 10 total number of required visits. From the optional modules, weight management was selected by most participants (72 [43%]), followed by managing stress (29 [17%]). Clinical teams were alerted to high blood pressure readings (from 68 participants) and other medical needs (from 64 participants). Participants received care from 75 unique VHA physicians at a given time. Some participants were assigned to 2 peer health coaches (23 [17%]), and the remaining participants were assigned to a single peer health coach (111 [83%]). There were 7 peer health coaches.

The trial participants had a mean (SD) age of 60.6 (9.7) years; included 229 males (87%) and 35 females (13%); self-identified as Hispanic (17 [6%]), non-Hispanic Black (73 [28%]), non-Hispanic White (123 [47%]), or multiracial (30 [11%]) individuals; and reported low annual income (103 [44%]). Participants across treatment groups were similar in age, race and ethnicity, annual income, and other demographic variables (Table 1). The mean (SD) SBP was 136 (18.0) mm Hg at baseline, and 40 participants (15%) had elevated blood pressure, 117 (44%) had stage 1 hypertension, and 66 (25%) had stage 2 hypertension. Almost all participants (242 [92%]) were in the overweight range (BMI ≥25). Overall, cardiovascular risk was high, as measured by the Framingham Risk Score (mean risk of a cardiovascular event in the next 10 years, 24%). Almost one-fifth of participants were current smokers (50 [19%]).

**Table 1.** Baseline Characteristics of Vet-COACH Trial Participants

Characteristic	Participants, No. (%)	
	Control group (n = 130)	Intervention group (n = 134)
**Demographic characteristics**		
Sex		
Male	116 (89)	113 (84)
Female	14 (11)	21 (16)
Age, mean (SD), y	60.9 (9.8)	60.3 (9.7)
Educational level: high school diploma	26 (20)	21 (16)
Employment status		
Married	47 (38)	57 (43)
Employed	47 (37)	53 (40)
Retired	49 (38)	48 (36)
Unable to work	21 (16)	16 (12)
Annual income: ≤$40 000	53 (45)	64 (52)
Race^a^		
Non-Hispanic Black	36 (28)	37 (28)
Non-Hispanic White	56 (43)	67 (50)
Multiracial	17 (13)	13 (10)
Hispanic ethnicity^a^	10 (8)	7 (5)
**Clinical characteristics and outcomes**		
SBP, mean (SD), mm Hg	135.1 (19.6)	137.1 (16.4)
DBP, mean (SD), mm Hg	81.3 (11.3)	80.9 (9.6)
Normal blood pressure, No. (%)	28 (22)	13 (10)
Elevated blood pressure: ≥120/80 mm Hg	16 (12)	24 (18)
Stage 1 hypertension: ≥130/90 mm Hg	52 (40)	65 (49)
Stage 2 hypertension: ≥140/90 mm Hg	34 (26)	32 (24)
FRS, mean (SD), %	0.26 (0.17)	0.22 (0.16)
Overweight: BMI ≥25	119 (92)	125 (93)
Obese: BMI ≥30	76 (59)	73 (55)
Current smoker	29 (22)	21 (16)
LDL-C, mean (SD), mg/dL	112.4 (38.7)	107.0 (38.6)
HRQOL MCS score, mean (SD), points	47.4 (11.9)	46.9 (11.7)
HRQOL PCS score, mean (SD), points	38.9 (10.6)	39.6 (10.6)
Hospitalizations in past year, mean (SD)	0.10 (0.37)	0.20 (0.52)
ED visits in past year, mean (SD)	1.21 (2.19)	1.03 (1.98)
Primary care visits in past year, mean (SD)	0.54 (1.27)	0.46 (1.15)

Abbreviations: BMI, body mass index (calculated as weight in kilograms divided by height in meters squared); DBP, diastolic blood pressure; ED, emergency department; FRS, Framingham Risk Score (10-year estimate of CVD risk, ranging from 0% to >30%); HRQOL, health-related quality of life; LDL-C, low-density lipoprotein cholesterol; MCS, SF-12 Mental Component Summary (range: 0-100 points, with lower scores indicating poorer mental HRQOL); PCS, Short Form 12 Item Physical Component Summary (range: 0-100 points, with lower scores indicating poorer physical HRQOL); SBP, systolic blood pressure; Vet-COACH, Veteran Peer Coaches Optimizing and Advancing Cardiac Health.

SI conversion factor: To convert LDL-C to millimoles per liter, multiply by 0.0259.

^a^ Race and ethnicity were self-identified by participants from the following categories: American Indian or Alaska Native; Asian and Pacific Islander; Black; Hispanic, Latino, or Spanish origin; non-Hispanic; White; or multiracial.

### Outcomes 

We found no significant difference in change in SBP between the intervention and control groups (−3.32 [95% CI, −6.88 to 0.23] mm Hg vs −0.40 [95% CI, −4.20 to 3.39] mm Hg; adjusted difference in differences, −2.05 [95% CI, −7.00 to 2.55] mm Hg; *P* = .40) (Table 2). We noted no difference in blood pressure control by number of peer health coach visits received (eTable 3 in Supplement 2). We found a significant improvement in change in HRQOL MCS scores in the intervention vs control group (2.19 [95% CI, 0.26-4.12] points vs −1.01 [95% CI, −2.91 to 0.88] points; adjusted difference in differences, 3.64 [95% CI, 0.66-6.63] points, *P* = .02). There were no differences in PCS scores, Framingham Risk Scores, or individual cardiovascular risks (BMI, low-density lipoprotein cholesterol level, and current smoking) (Table 2).

**Table 2.** Differences in Primary and Secondary Outcomes Between Intervention and Control Groups

Change measure	Difference (95% CI)		Intervention effect	
	Control group (n = 130)	Intervention group (n = 134)	Adjusted difference in differences (95% CI)	*P* value
SBP, mean (SD), mm Hg	−0.40 (−4.20 to 3.39)	−3.32 (−6.88 to 0.23)	−2.05 (−7.00 to 2.55)	.40
DBP, mean (SD), mm Hg	−0.37 (−2.68 to 1.95)	−0.64 (−2.71 to 1.42)	−0.81 (−3.92 to 2.12)	.60
% BP ≤120/80, OR (95% CI)	NA	NA	1.40 (0.64 to 3.04)	.41
Change in FRS, mean (SD), %	−0.01 (−0.03 to 0.004)	−0.01 (−0.03 to 0.004)	−0.01 (−0.03 to 0.01)	.45
Change in BMI, mean (SD)	0.10 (−0.3 to 0.5)	−0.04 (−0.48 to 0.40)	−0.20 (−0.96 to 0.50)	.59
LDL-C, mean (SD), mg/dL	−4.24 (−9.75 to 1.27)	−3.12 (−8.36 to 2.12)	−0.56 (−7.60 to 6.57)	.88
Current smoker, OR (95% CI)^a^	NA	NA	0.92 (0.22 to 3.79)	.90
Change in HRQOL MCS score, mean (SD), points ^b^	−1.01 (−2.91 to 0.88)	2.19 (0.26 to 4.12)	3.64 (0.66 to 6.63)	.02
Change in HRQOL PCS score, mean (SD), points ^b^	0.72 (−0.82 to 2.27)	1.02 (−0.73 to 2.77)	0.51 (−1.76 to 2.77)	.66

Abbreviations: BMI, body mass index (calculated as weight in kilograms divided by height in meters squared); BP, blood pressure; DBP, diastolic blood pressure; FRS, Framingham Risk Score (10-year estimate of CVD risk, ranging from 0% to >30%); HRQOL, health-related quality of life; LDL-C, low-density lipoprotein cholesterol; MCS, Mental Component Summary (range: 0-100 points, with lower scores indicating poorer mental HRQOL); NA, not applicable; OR, odds ratio; PCS, Physical Component Summary (range: 0-100 points, with lower scores indicating poorer physical HRQOL); SBP, systolic blood pressure.

SI conversion factor: To convert LDL-C to millimoles per liter, multiply by 0.0259.

^a^ Smoker results are logistic regression.

^b^ The PCS score had more than 15% missing data; results were based on multiple imputation analysis.

We did not find a significant difference in number of hospitalizations, ED visits, or primary care visits in the 12 months after randomization among the intervention vs control participants (Table 3). The analyses of outcomes in the post–COVID-19 period resulted in similar results as the full analysis except for the hospitalization model, which we were unable to run due to the small number of events (eTables 4-6 in Supplement 2).

**Table 3.** Previous Year Health Care Use at Baseline and 12 Months Between Intervention and Control Groups

Measure	Control group (n = 130)		Intervention group (n = 134)		Adjusted difference in differences (95% CI)	*P* value
	Baseline	12-mo	Baseline	12-mo		
Outpatient visits in past year, mean No. (SD)	0.54 (1.27)	0.55 (1.35)	0.46 (1.15)	0.40 (0.89)	−0.093 (−0.279 to 0.146)	.40
ED visits in past year, mean No. (SD)	1.21 (2.19)	0.86 (1.66)	1.03 (1.98)	0.86 (1.37)	0.018 (−0.306 to 0.403)	.93
Hospitalized in past year, mean No. (SD)	0.10 (0.37)	0.13 (0.47)	0.20 (0.52)	0.16 (0.53)	−0.076 (−0.191 to 0.122)	.35

Abbreviation: ED, emergency department.

## Discussion

The goal of the Vet-COACH trial was to test a peer-support intervention aimed at improving cardiovascular health and quality of life among veterans who resided in areas with a high prevalence of hypertension. We used a novel neighborhood-based strategy and recruited a racially diverse population with low annual income. We found that the peer health coaching intervention did not improve blood pressure levels but led to improvements in mental HRQOL, as measured by the SF-12 MCS score, which included questions about impact on emotions, ability to function, pain interfering with work, energy level, calmness, and depression. The difference of 3.64 points exceeded the minimally important difference (ie, smallest change in an outcome that a patient would identify as important) of 3 for the SF-12 MCS score,^20^ suggesting that changes observed in the intervention group have meaningful implications for veterans. This threshold also corresponded to an effect size of approximately 0.5, which is consistent with a moderate effect based on the Cohen *d* criteria. This effect size has been identified as the minimally important difference for changes in HRQOL among individuals with chronic diseases, regardless of the HRQOL measure, disease, and minimally important difference estimation method.^30^ This effect may be especially important for veterans, who have worse health status than the general population,^31^ and for the study sample, whose HRQOL scores were lower than the national mean (50 points).

Although disproportionately affected by CVD, Black adults remain underrepresented in clinical trials.^30^ In contrast, we were able to recruit a substantial number of Black participants and peer health coaches.^16^ The sample included 28% Black veterans, a substantially higher proportion than that in national estimates (15% Black veterans)^32^ of VHA users and in clinic populations (based on administrative data). The neighborhood-based recruitment strategy for both participants and peer health coaches identified Census tracts with the highest prevalence of hypertension within the patient population.^16^ Census tracts closely approximate neighborhoods and are relatively homogeneous with respect to population characteristics, economic circumstances, and living conditions.^33^ We identified Census tracts with high rates of hypertension that mapped closely to areas of high social vulnerability and poverty,^16,34^ suggesting a reproducible method that health systems could use for disease prevention strategies or that researchers could use for recruiting at-risk patient populations.

### Strengths and Limitations

The Vet-COACH trial has several strengths. First, intervention protocols were designed to be practical and feasible, thus allowing replication in other clinic settings. Second, we accounted for clustering by peer health coach. Largely ignored in peer health coaching studies is the peer health coach’s contribution to the intervention effect.^27^ Success of the intervention could depend on the skill with which it is delivered, and ignoring this factor could lead to bias in treatment effects and CIs. Third, we used a novel area-based strategy that enabled us to recruit a racially diverse population with low income who were most affected by cardiovascular risks.

This study also has several limitations. First, due to a variety of challenges, including the COVID-19 pandemic, the trial was underpowered to detect a difference in the primary outcome due to lower-than-projected sample size.^16^ Similar to other studies,^10^ this trial’s baseline enrollment data suggested that many participants had already received appropriate treatment for their elevated blood pressure, decreasing the power to show a statistically significant change in SBP. The decision to enroll participants with adequate blood pressure control at baseline may have contributed to the null effect.^35^ Additionally, reporting control participants’ elevated blood pressure to the primary care teams may have biased the results to the null, as this reporting may have prompted clinical action. There is also the possibility of misclassification of blood pressure due to inaccurate readings. Second, despite our multiple attempts to complete exit visits, 15% of the participants were lost to follow-up, although we were able to obtain blood pressure readings from the electronic health record data from participants who did not schedule an exit visit. Third, the trial took place in 1 geographic area of the US, which may affect generalizability. Internal validity may be affected by the inability to blind participants or study staff to the intervention. Fourth, some outcomes were limited to self-report (eg, smoking status, physical activity, and dietary intake); self-report is subject to recall and other biases.

## Conclusions

The Vet-COACH randomized clinical trial adds to the literature on the effectiveness of peer support, which has shown mixed results,^31,36,37^ including a meta-analysis of peer-support interventions that reported an overall modest, but not clinically significant, change in blood pressure of 2.07 mm Hg for patients with diabetes and hypertension.^38^ The results of the present trial showed a benefit in targeting HRQOL, an important patient-reported outcome, and were consistent with other peer-support studies that reported quality-of-life^39^ benefits and suggested that peer health coaching may create opportunities for well-being improvements beyond blood pressure control.^40^ Peer support falls into the social support model, which posits that social relationships improve health behaviors, HRQOL, and well-being.^39^ Among veterans, the peer support model may be especially effective due to the shared military experience and camaraderie of veterans. Providing social support via veteran peer health coaches of similar socioeconomic and military backgrounds who live in the same neighborhoods may be one model that can improve patient HRQOL.

## Notes

Supplement 1.
Trial Protocol

Supplement 2. **eMethods.** Multiple Imputation of MCS and PCS **eTable 1.** Baseline Characteristics of Trial Participants by Missing SF-12 Status **eTable 2.** Change in MCS/PCS Between VetCOACH Intervention and Control Groups Using Completers Vs. Multiple Imputation **eTable 3.** Distribution of Completion of Core Visits Across Study Participants **eTable 4.** Pre-/Post-Covid Differences in Primary and Secondary Outcomes Between VetCOACH Intervention and Control Groups **eTable 5.** Pre-Covid Health Care Use Outcomes **eTable 6.** Post-Covid Health Care Use Outcomes

Supplement 3.
Data Sharing Statement
