# Supplementary material for: Effect of a Peer Health Coaching Intervention on Clinical Outcomes Among US Veterans With Cardiovascular Risks: The Vet-COACH Randomized Clinical Trial
Source: JAMA Netw Open. 2023 Jun 6;6(6):e2317046. doi: 10.1001/jamanetworkopen.2023.17046 (PMC10245194; doi:10.1001/jamanetworkopen.2023.17046)
Supplement: Supplement 1. — Trial Protocol [file jamanetwopen-e2317046-s001.pdf]

Human Subjects Protocol

VA Puget Sound IRB

IIR 14-063

Vet-COACH (Veteran peer Coaches Optimizing and Advancing Cardiac Health)

MIRB #: 00826

HSR&D

Principal Investigator: Karin M. Nelson, MD MSHS

[V2]

Abstract

BACKGROUND: Cardiovascular disease (CVD) is the leading cause of mortality among Veterans, and suboptimal risk factor control is an important mechanism for the continued prevalence of CVD. Despite clinic based programming that includes nurse care management, pharmacy support, telephone care programs and intensive quality improvement efforts, CVD risk factors remain sub-optimally controlled among Veterans. Given the high prevalence and cost CVD within the Department of Veterans Affairs, cost-effective mechanisms are needed to manage the burden of prevalent CVD risk factors. Veteran peer health coaches may be one such mechanism; however, previous work has provided limited data of this model with VHA primary care. Previous studies of peer support in non-VHA populations report significant improvement in hypertension control and CVD risk reduction.

OBJECTIVES: The proposed project will test the effectiveness of **Vet-COACH** (**Vet**eran peer **C**oaches **O**ptimizing and **A**dvancing **C**ardiac **H**ealth), a peer health coaching program to help reduce CVD risk among Veterans. The overall goal of this study is to test the effectiveness of a peer health coach intervention to promote health behavior change among Veterans with multiple CVD risk factors with a hybrid type 1 implementation study. To target a high risk population, we will focus on Veterans with poorly controlled hypertension and at least one other CVD risk factor.

METHODS: We will conduct a randomized controlled trial that will enroll n=400 Veterans to compare a peer health coach intervention consisting of telephone support, and linkages to appropriate community-based and clinic resources compared to usual VHA primary care. The primary outcome is reduction in systolic blood pressure from baseline to follow-up at 1-year. Secondary outcomes include a reduction in Framingham Cardiovascular risk score, individual cardiovascular risks (tobacco use, lipids), health related quality of life and health care use. We will also assess the effects of the peer health coach intervention on intermediate outcomes including social support, patient activation, patient/provider communication and health behaviors (e.g. medication adherence, physical activity, nutrition, alcohol use, and stress management). In addition, we will assess the cost of the intervention to inform feasibility for future studies, determine Veteran and staff satisfaction with the intervention, and identify barriers and facilitators to adoption.

IMPACT ON VETERANS HEALTH CARE: Integrating peer health coaches into PACT primary care teams may improve the VA's ability to provide community outreach to Veterans. CVD risk reduction provides an ideal target for intervention given the prevalence of modifiable risks among Veterans. The proposed study will increase understanding of the utilization of peer support within PACT teams. If this study proves the main hypothesis, this evidence-based support model could be tested more widely among Veterans with other chronic conditions to improve health outcomes.

List of Abbreviations

AHA	American Heart Association
ALVA	American Lake VA
AUDIT-C	Alcohol Use Disorders Identification Test
BMI	Body Mass Index
BRFSS	Behavioral Risk Factor Surveillance Survey
CAHPS	Consumer Assessment of Health Plans Study
CDW	Corporate Data Warehouse
CHW	Community Health Worker
CVA	Cardiovascular Accident
CVD	Cardiovascular Disease
DUA	Data Use Agreements
FFQ	Food Frequency Questionnaire
FRS	Framingham Risk Score
GIS	Geographic Information Systems
HLD	Hyperlipidemia
HPDP	Health Promotion and Disease Prevention
IHD	Ischemic Heart Disease
IPAQ	International Physical Activity Questionnaire
LDL-C	Low Density Lipoprotein
MOU	Memoranda of Understandings
MOVE	Weight Management Program for Veterans
OMHS	Office of Mental Health Services
PACT	Patient Aligned Care Team
PAM	Patient Activation Measure
PTSD	Post-traumatic stress disorder
PVD	Peripheral Vascular Disease
SBP	Systolic Blood Pressure
Vet-COACH	Veteran peer Coaches Optimizing and Advancing Cardiac Health
VFW	Veterans of Foreign Wars

Contents

Protocol Title: 5
1.0 Study Personnel 5
2.0 Introduction 5
3.0 Objectives 8
4.0 Resources and Personnel 10
5.0 Study Procedures 11
5.1 Study Design 11
5.2 Recruitment Methods 16
5.3 Informed Consent Procedures 17
5.4 Inclusion/Exclusion Criteria 18
5.5 Study Evaluations 19
5.6 Data Analysis 20
5.7 Withdrawal of Subjects 26
6.0 Reporting 26
7.0 Privacy and Confidentiality 27
8.0 Communication Plan 28
9.0 Information Security & Privacy 29
10.0 References 28

Protocol Title:

1.0 Study Personnel (KEY)

- Provide name, contact information, and affiliations/employee status for the following:

Principal Investigator:

Karin M. Nelson, MD MSHS
Karin.nelson@va.gov 206.277.4507
Core Investigator/Assoc. Professor of Medicine
VA Puget Sound Health Care System
1660 S. Columbian Way, Seattle, WA 98108

Co-Investigators:

Tiffanie Fennell, PhD
Tiffanie.fennell@va.gov 206.277.4434
Clinical Health Psychologist, Health Behavior Coordinator
VA Puget Sound Health Care System
1660 S. Columbian Way, Seattle, WA 98108

Collaborators (at other institutions, not covered under the VA IRB approval):

None

2.0 Introduction

- Provide scientific background and rationale for study.
- Include summary of gaps in current knowledge, relevant data, and how the study will add to existing knowledge.
- Include rationale for including or excluding certain populations – in particular vulnerable populations.

High prevalence and sub-optimal control of CVD risk factors among Veterans:

Cardiovascular risk is a composite measure of several modifiable risk factors including hypertension, dyslipidemia, and tobacco use. Sedentary lifestyles, unhealthy diets, and increasing obesity rates will likely continue to increase rates of hypertension, dyslipidemia and CVD.[18-20] Because of these trends, a 2020 American Heart Association (AHA) goal is the promotion of ideal cardiovascular health, defined as the presence of 4 health behaviors (non smoking, BMI ≤ 25 mg/kg^2, physical activity at goal level, and diet consistent with current guidelines) and the presence of at least 2 ideal health factors (untreated total cholesterol < 200 mg/dl, untreated blood pressure < 120/80 mmHg, or untreated fasting glucose < 100 mg/dl).[4] The majority of U.S. adults meet very few of these goals.[21,22] Similar to the US population, Veterans have high rates of CVD risks.[2] Almost half of Veterans seen in primary care have a hypertension diagnosis and one quarter have poor control.[23] Veterans who obtain care from VHA are more likely to be obese and physically inactive than the general population[5] and the prevalence of tobacco use among Veterans is 25%.[6]

CVD risk factor control will require a multi-faceted intervention:

Comprehensive approaches to control CVD risk will need to focus on multiple risk factors including blood pressure, high cholesterol, tobacco use, body-mass index, physical activity and nutrition.[24,25] Control of hypertension and other modifiable CVD risks requires medication management in addition to behavioral interventions to change health behavior. National guidelines recommend that all individuals with hypertension undergo the following lifestyle modifications: loss of 5-10% of body weight, or maintain normal weight; moderate physical activity, change diet to be low sodium; smoking cessation and moderate alcohol consumption.[26] Focusing on more than one of these aspects of healthy lifestyle is synergistic and can lead to greater improvements in hypertension control.[27] Existing studies suggest significant benefit in blood pressure control from lifestyle modifications[26,28,29] with a decrease in SBP from 5 - 20 mm Hg with 10 kg loss of body weight, a decrease in SBP from 8 - 14 mm Hg with dietary changes,[30] a decrease of 4 - 9 mm Hg with regular physical activity[31,32] and 2 - 4 mmHg with moderate alcohol consumption.[33] Given the high rates of medication non-adherence among individuals with hypertension,[34] focusing on this topic is critical.

Gaps in knowledge about peer support interventions among Veterans in primary care:
Trained peers may be a novel and innovative addition to existing programs, as peers can be effective in supporting health behavior change[7] and make healthcare more patient-centered.[41,42] Peer support, defined as the provision of support and information from a member of a social group who possesses experiential knowledge and similar characteristics to the target population, falls into the social support model, where social relationships improve health-related quality of life and well-being.[43] To date, peers have been utilized in several studies at VHA, including two studies that showed significant clinical improvement among patients with diabetes.[12,13] Previous research in non-VA populations suggests that peer coaches working in conjunction with nurses are effective at controlling cardiovascular risk factors,[8,10,44] but work is needed to determine how to integrate peer support into PACT teams to help assist Veterans. One component of the PACT initiative is the utilization of RN care managers to coordinate care. Care management provided by registered nurses can be effective, but is expensive. Veteran peer health coaches are a potentially more cost effective mechanism to increase outreach and link Veterans to their PACT team and RN care managers.

Peer support and the unique experiences of the Veteran community:

The positive health benefits of peer support may be particularly strong in the Veteran community. Veterans have long organized into community organizations (e.g. VFWs) based on

their unique military experience, the powerful formative experiences of military service and unique post-discharge experiences (e.g. PTSD, integrating back into civilian life). Because of these common experiences, Veterans may find it easier to understand and accept the health recommendations of a peer health coach.[45] Peer coaches may also be able to help Veterans and providers to understand each other's perspective and priorities. This shared experience has been utilized effectively in the treatment of Veterans with mental health conditions. The VHA Office of Mental Health Services (OMHS) has a well-developed peer support program and has hired 300 Veterans to assist mental health clinical teams. The core features of the recovery model for mental health (e.g. peer support, holistic orientation, self-direction, and patient-centeredness) are integral to most chronic disease self-management programs.[15] However, the peer support approach has not received widespread testing among Veterans cared for in primary care. By leveraging the shared military experience and camaraderie of Veterans, the peer coach model may be an untapped opportunity to improve health among primary care patients and may work more effectively in the VA, but this model needs further testing.

Contributions of the proposed work:

CVD remains the number one cause of mortality among Veterans and sub-optimal risk factor control contributes to the ongoing prevalence of CVD. Our goal is to decrease CVD risk among Veterans by addressing modifiable risk factors (hypertension, elevated cholesterol, overweight/obesity and tobacco use) with an innovative, patient-centered intervention. Our approach to multiple risk reduction with a novel peer coach intervention mirrors clinical practice, where multiple conditions and behaviors are considered simultaneously. Peer support models have been utilized effectively in other patient populations to manage CVD risk and improve BP control, but limited work has been done within the context of the PACT team or in Veteran populations. The intervention will be integrated into primary care clinics and PACT teams at two clinical sites, enhancing the potential for benefit and generalizability to other settings. The intervention is consistent with the PACT goals to provide more longitudinal and less episodic care.[16] The proposed intervention could potentially decrease CVD risk among Veterans by 1) building upon previous work with peer support among non-Veteran populations; 2) focusing on multiple synergistic CVD-related behaviors and risk factors and 3) targeting a high risk population (e.g. Veterans with 2 or more CVD risks) thereby providing the greatest potential to improve morbidity and mortality. Our proposal is innovative in the use of a hybrid type 1 design, which will inform future implementation efforts. The Vet-COACH intervention was designed with our clinical partners and is integrated into the PACT team. Given the national prevalence of CVD and the low rates of risk factor control, easily disseminated interventions such as the one proposed could significantly improve health outcomes.

Relevance to VA Care:

Given the numbers of Veterans with sub-optimal cardiovascular control, developing models of care to best help this population is critical to the VHA mission. Peer support may provide a needed link between Veterans and available VHA clinical services and serve as a community-clinic liaison to strengthen connections to community-based resources. Utilizing trained peers may be one mechanism to improve health among Veterans. However, much research is needed to determine the role and effectiveness of peer support. The VHA has a well-developed evidenced-based Health Promotion and Disease Prevention (HPDP) program. Expanding the reach of this program by the use of peer health coaches has the potential to provide cost-effective health coaching.

Unique and innovative features of the Vet-COACH intervention include:

- Veterans will not have to travel to a class or clinic, thereby increasing participation in self-management education, enhancing recruitment and retention.

- Veterans will be more comfortable in their homes during the health coaching calls, and therefore more able to participate actively in learning activities and develop trusting relationships with the Veteran peer health coaches.
- Peer health coaches will be able to role-model and observe participants in practicing self-management behaviors (e.g. managing medications).
- Peer health coaches will be able to make suggestions that household members be enlistedto provide support for Veterans.
- Peer health coaches will be able to link Veterans to pre-existing programs (e.g. disease self-management classes, MOVE, home tele-monitoring).
- Peer health coaches have access to PACT teams and can assist Veterans engagement in care.

The proposed research has the potential to contribute to the VHA mission to provide patient centered care, and also to develop, facilitate and integrate peer support into PACT teams. This proposal directly targets improving a Veteran-centric health care model to help Veterans navigate the health care delivery system and receive coordinated care. By providing training curricula and data on the effectiveness of peer health coaches, our goal is to present VHA policymakers with evidence regarding peer health coaches work to decrease CV risk and improve Veteran health.

3.0 Objectives

- Describe the study's purpose, specific aims, or objectives.
- State the hypotheses to be tested.

Cardiovascular disease (CVD) is the leading cause of mortality among Veterans, and sub-optimal risk factor control is an important mechanism for the continued prevalence of CVD.[1,2] Despite clinic-based programming that includes nurse care management, pharmacy support, telephone care programs and intensive quality improvement efforts, CVD risk factors remain sub-optimally controlled among Veterans.[3] Modifiable CVD risks include hypertension, hyperlipidemia (HLD), obesity and tobacco use. These conditions are common, often co-exist, and are amenable to behavior change and disease self-management interventions.[4] Among Veterans with hypertension, 25% have poor control (as defined by a blood pressure of >140/90 mmHg). Similar to the US population, the majority of Veterans are overweight or obese, are sedentary and eat unhealthy diets.[5] Over one quarter of Veterans smoke.[6] To improve health and decrease CVD morbidity and mortality, CVD risk reduction is an important issue facing VHA and other U.S. health systems.

There is a growing literature about the positive health benefits of peer support on health outcomes and health behavior change.[7] Previous studies of peer support in non-VHA populations report significant improvement in hypertension control and CVD risk reduction.[8-11] There are several peer support titles, including peer coaches, navigators, and community health workers (CHWs), with the common characteristic of sharing life experiences and social background with the patients being served, and promoting health by providing education, assistance, motivation and social support.[7] Several models of peer support have been tested in

VHA, but have primarily targeted single conditions, e.g. diabetes[12,13] or among Veterans with mental health conditions.[14,15]

Specific Aims:

1. Test the effectiveness of a peer health coach intervention for improving health outcomes for Veterans with multiple CVD risks in a randomized controlled trial. The primary outcome is a reduction in systolic blood pressure (SBP) from baseline to follow-up at 1 year. Secondary outcomes include change in cardiovascular risk, as measured by the Framingham Risk Score[17], other cardiovascular risks (tobacco use, lipids) and health related quality of life. Using administrative data at 1 year following randomization, we will examine healthcare utilization, hospitalizations, and emergency room visits.
2. Assess the effects of a peer health coach intervention on intermediate outcomes that are intervention targets including social support, patient activation, *patient/provider communication* and health behaviors (e.g. medication adherence, physical activity, nutrition, *alcohol use, and stress management*).
3. Assess the cost of the intervention to inform feasibility for future studies.
4. Determine Veteran and staff satisfaction with the intervention and identify barriers and facilitators to adoption.

The recent VHA medical home model initiative, the Patient Aligned Care Team (PACT), is an ideal setting to test peer support, as this social support model aligns with the PACT focus on team-based, patient-centered, and comprehensive care.[16] The PACT team includes a primary care provider, an RN care manager, and a LPN; as well as support staff including pharmacists and Health Promotion and Disease Prevention (HPDP) staff. The focus of the HPDP program is to support chronic disease self-management and promote healthy lifestyle choices. **Trained Veterans working as peer health coaches may be a mechanism to expand the reach of the PACT team by (1) providing brief health education messages based on HPDP program materials, (2) acting as a community-clinic liaison to create stronger linkages between primary care and community resources and (3) by linking Veterans to community-based resources for healthy lifestyle choices.** The overall goal of this study is to test the effectiveness of a peer health coach intervention (**Vet-COACH** "**Vet**eran peer **C**oaches **O**ptimizing and **A**dvancing **C**ardiac **H**ealth") to promote health behavior change among Veterans with multiple CVD risk factors with a *4*-year hybrid type 1 implementation study**.** To target a high risk population, we will focus on Veterans with poorly controlled hypertension and at least one other CVD risk.

Integrating peer health coaches into the PACT primary care team may improve the VA's ability to provide community outreach to Veterans. CVD risk reduction provides an ideal target for intervention given the prevalence of modifiable risks among Veterans. The proposed study will increase understanding of the utilization of peer support within PACT teams. If this study proves the main hypothesis, this evidence-based support model could be tested more widely among Veterans with other chronic conditions to improve health outcomes.

4.0 Resources and Personnel

- Include where and by whom the research will be conducted.
- Provide a brief description of each individual's role in the study. Be sure to indicate who will have access to protected health information and who will be involved in recruiting subjects; obtaining informed consent; administering survey/interview procedures; and performing data analysis.
- If applicable provide information on any services that will be performed by contractors including what is being contracted out and with whom.

  N/A

- If applicable provide information on any Memoranda of Understandings (MOUs) or Data Use Agreements (DUAs) that are being entered into including with whom and for what reason.

  N/A

  Research will be conducted at VA Puget Sound Health Care by the Vet-COACH project study staff:

Name	Role	Access to PHI	Recruitment/Informed Consent/Surveys/Interviews	Data Analysis
Karin Nelson	PI	Y	Y	Y
Tiffanie Fennell	Co-I	Y	N	Y
Leslie Taylor	Biostatistician	N	N	Y
George Sayre	Qualitative Analyst	Y	N	Y
Marie Lutton	Project Manager	Y	Y	N
Jennifer Williams	Research Assistant	Y	Y	N
Jeff Rodenbaugh	Programmer	Y	N	Y
Kristen Gray	Analyst	Y	N	Y
Matt Augustine	Analyst	Y	N	Y
Kristin Rosendahl	Nutrition expert	Y	N	N
Brittney Hamilton	Research Assistant	Y	Y	N
Charles Bradley Kramer	Graduate Research Student Assistant (RA)	Y	Y	Y
Elizabeth Presley Strewler	Research Social Worker / Research Coordinator	Y	Y	N

5.0 Study Procedures

5.1 Study Design

- Describe experimental design of the study. Include sequential and/or parallel phases of the study, including durations, and explain which interventions are standard of care.
- Include a description of how anticipated risk will be minimized and include an analysis of risk vs. potential benefit.
- Provide description of the study population (delineate all categories of subjects – patients, providers, family members, employees, etc.). Include anticipated enrollment numbers
- As applicable, provide information on any added protections for vulnerable populations.
- If applicable include information on data and specimen banking.

Study design overview:

We propose a randomized controlled trial to evaluate the effectiveness of **Vet-COACH**, a peer health coach program to control hypertension and reduce CVD risk among Veterans. The original program incorporated home visits by peer health coaches to deliver health education, counseling and goal setting support. Due to safety concerns and social distancing restrictions stemming from the COVID 19 pandemic, the program has transitioned to conducting all health coaching encounters, as well as enrollment and exit visits, via phone. This transition was implemented under IRB approval of the miscellaneous memo submitted at the beginning of the pandemic. These changes to conduct all health coaching encounters and exit surveys over the phone will be permanent for the remainder of the study. Our operations partners from NCP have developed extensive patient educational materials that will be used for the current study.[55] We will train the peer health coaches to provide brief educational messages and link Veterans to existing clinical services at VHA, including their primary care PACT teams and community based programs. The main outcome will be a reduction in systolic blood pressure. Secondary outcomes are cardiovascular risk, as measured by the Framingham Risk Score (FRS),[17] health care utilization and intermediate behavioral outcomes including patient activation, social support and health behaviors (e.g. diet and physical activity, medication adherence). *We will randomize a total of 200 individuals to the intervention group and 200 individuals to the control group*.

Our study design has limitations. Because of the nature of the intervention, we are unable to blind treatment assignment. The PI and Dr. Fennell will provide overall supervision to the peer coaches. This model is necessary for program development. Because the intervention is being conducted at two sites, we are not proposing an economic evaluation to estimate cost-effectiveness, in part because such data would be of limited generalizability. Positive findings will lead to a larger multi-site trial that would include a cost-effectiveness analysis. These limitations are more than balanced by the strengths of this proposal. The proposed project is a prospective, randomized controlled trial that examines important outcomes with statistical power to detect clinically meaningful differences.

Description of Vet-COACH – a call-based peer health coach intervention:

The three goals of the peer health coach intervention include: (1) delivering brief health messages based on materials from the HPDP program, (2) acting as a community-clinic liaison and (3) providing expertise on the availability of community based resources for healthy lifestyle choices (e.g. places for physical activity, walking groups, grocery store tours, etc.) **The focus of the intervention will be the provision of brief health messages, goal setting and action planning around health behavior changes shown to decrease CVD risk** (e.g. healthy diet, regular moderate-intensity physical activity, tobacco cessation if applicable). Peer health coach education modules, examples of key messages and measures used to assess participant progress are outlined in Table 2. Required education modules for hypertension include: physical activity, nutrition, and medication adherence (and weight management if overweight or obese).[56]

Table 2. Veteran Peer Health Coach Required Education Module Topics, Examples of Goals and Key Messages, Referrals to Community or VHA resources, and Measures Used to Assess Intermediate Outcomes

**Behavioral targets**	Goals and Key Messages	Referral to appropriate community or VHA resources	Intermediate outcome measures
**Required modules – applies to all Veterans with Hypertension**			
**Blood Pressure**	What is high Blood Pressure? Understand systolic & diastolic measurements. Tips to manage blood pressure.	Offer blood pressure cuff if subject does not have one. Record blood pressure measurements & report to PI, study nurse and/or PCP as needed.	Standard protocols
**Physical activity**	Avoid inactivity. Every 10 minute session of physical activity counts. Goal of moderate exercise for at least 30 minutes per day.	List of local physical activity resources provided to all participants<br>Offer pedometers	IPAQ[57]
**Nutrition**	Healthy plate concept. Areas of focus will include reducing portion size, reducing fast food intake, avoiding processed foods high in sodium and carbohydrates, reduction of salt intake, increase in fruit and vegetable intake and decrease in total and saturated fat.	Encourage referral to VHA nutrition<br>Offer healthy plate<br>Grocery store tour<br>Information on local resources (e.g., food banks, farmers markets)	Self-reported nutrition behavior[57] and food frequency (FFQ)[58]
**Medication adherence**	Know your medications and which ones you take. Have a written list and bring to each health care visit.	Feedback to PCP, nurse case manager<br>Offer medication organizer	Self-reported medication adherence (Voils, C. I. et al.)[59]<br>ReCOMP[60]
**Communication with medical team/physician**	Be involved with your health care. There are many ways to take an active role.	Feedback to PCP, nurse case manager for appropriate follow up	CAHPS-provider communication[63]
**Optional Education modules- if applicable to individual Veteran**			

**Weight reduction, maintenance of normal BMI**	Strive for a healthy weight. If you need to lose weight, even a small amount can improve your health. If you are a normal weight, maintain it.	Referral to MOVE program, assistance with enrollment questionnaire (MOVE23)	BMI
**Smoking cessation**	Be tobacco free! If you are using tobacco, ask your health care team for help in quitting.	Referral to smoking cessation resources, provide quit line number	Self-report of smoking status<br>New medication for smoking cessation
**Limiting alcohol intake**	If you choose to drink, do so in moderation. If drinking, limit to <= 2 drinks/day for men, <= 1 drink for women	Feedback to PCP, nurse case manager for appropriate follow up	AUDIT-C[61]
**Stress management, depression and sleep**	Pay attention to stress. Tools are available to help you manage and reduce stress.	Feedback to PCP, nurse case manager for appropriate follow up;<br>Referral to Mindfulness based stress reduction class	PHQ-8[62]

Participant Inclusion criteria:

We will focus our intervention in the geographic area directly surrounding the VA Puget Sound (Seattle and ALVA clinics) that has a high prevalence of poorly controlled hypertension. As part of project planning, we have worked with the Office of Analytics and Business Intelligence to utilize Geographic Information Systems (GIS) to identify and target high risk areas. This type of patient mapping is available nationwide, so this mechanism to identify and recruit participants is potentially generalizable.

We developed our inclusion criteria to identify Veterans who will benefit the most, by focusing on Veterans up to age 75 years with poorly controlled risks that are amenable to change. Specific inclusion criteria include poorly controlled hypertension (mean systolic BP > 150/90 mmHg) and one or more of the following CVD risks: overweight or obese (Body Mass Index (BMI) $\geq$ 25 kg/m^2), current tobacco use, or a diagnosis of hyperlipidemia (defined as low-density lipoprotein (LDL-c) >130 mg/dL). Although we are selecting common conditions that increase cardiovascular risk, we are not excluding Veterans with other prevalent conditions (e.g. diabetes, arthritis, renal disease, or COPD), whose presence will be noted in the enrollment visit. We chose to focus on Veterans with elevations in systolic hypertension above the stage 1 level of 140 mmHg, given recent data regarding treatment of mild hypertension.

Exclusion criteria:

We will exclude those who have been hospitalized 3 months prior to enrollment for a CVD admission (IHD, CVA or PVD), as these Veterans may be too ill to participate in this intervention. We will exclude patients with dementia or severe cognitive impairment that would not allow them to participate in the program; patients with a suicidal ideation and/or disruptive behavior flag in CPRS; patients with end stage renal disease on dialysis, given the unique care

needs of this population; patients who have received palliative care or are enrolled in hospice care, given their limited life expectancy; Veterans enrolled in Home Based Primary Care, an all-inclusive home visit nurse program or those who live in care facilities (nursing homes, assisted living facilities); homeless Veterans and women who are pregnant or planning to become pregnant.

Enrollment and randomization:

a) Enrollment and exit visits

The research coordinators will interview participants during the enrollment and exit visits over the phone. If a participant expresses completion of the exit survey over the phone would pose difficulties for them, staff will provide the option to complete and return the exit survey by mail. If the participant agrees, staff will mail a copy of the exit survey with a postage-paid return envelope to mail the completed survey to the HSR&D office. Study staff in possession of a VA-issued flip phone will use it to conduct the call. Study staff who do not have a VA phone or laptop will use their personal smart phone and conduct the call using the Doximity app. Scheduled participants will be mailed a letter prior to their enrollment visit that will confirm the date, time, and location of their appointment and a brief summary of what will be covered in the visit and program. The baseline enrollment visit and exit call will include blood pressure measurements and labs (lipids). If there are no lipid panel results in CPRS dated within 12 months prior to the baseline/enrollment date or 3 months prior to the exit visit date, the PI will order a lipid panel test. If a participant moves out of the Puget Sound area and/or receives lipid test results from a non-VA provider or clinic, we will request they send the lipid panel test results from the non-VA lab or VA lab in another state to the Puget Sound VA Primary Care Clinic via mail or secure fax. This is already done by VA patients who may get bloodwork at a non-VA facility. These results would be used as the lipid measurements typically collected at the exit visit. Juliana Bondzie (study coordinator and phlebotomist) will conduct the blood draw with participants completing the enrollment and/or exit visit with her. Ms. Bondzie has completed all the required trainings and coursework to conduct blood draws at the VA Puget Sound. Any results flagged as high will be reported by the PI to the participant's PCP -- either as an addendum to the research enrollment note or as an alert to the PCP if they are not enrolled in the study -- using the following statement: "We ordered a lipid panel test as part of screening or follow up for the Vet-Coach research project. Your patient may or may not participate in this study." Three blood pressure measurements will be collected by the research coordinators at intervals during the enrollment call. For BP measurement, after the patient has sat comfortably for 5 minutes, three BP consecutive measures are obtained and then averaged per NHANES protocol. [57]All BP measurements will be performed with electronic, upper arm cuffs.[58] The average BP must be >150/90 mm Hg at the time of the data pull for Veterans to be eligible for the study. We will attempt to schedule enrollment visits within a week of their most recent primary care visit to recruit Veterans with uncontrolled hypertension. Height and weight will be measured at baseline and weight will be measured at exit. Measures collected include: chronic disease self-management behaviors, health history, health care access, and health care utilization. Urgent issues will be referred immediately to the PI. The PI will contact the PCP for urgent issues to ensure appropriate follow-up.

b) Randomization

Following baseline assessments, a computerized randomization procedure will assign individuals to either the peer health coach intervention or to the usual care + education control group. Randomization of participants will occur in the order of completed enrollment visits. Randomization lists will be constructed in advance by the study biostatistician using variable

length blocks to ensure the groups are not unbalanced at any time. Concealment will be used to prevent the study staff from obtaining information on the sequence of assignment.

c) Usual care + education control arm

Usual care consists of enrollment in the Primary Care Clinic or Women's Clinic at VA Puget Sound, plus an educational pamphlet with study materials provided at the baseline visit. The educational pamphlet will deliver the same information content as the intervention and consist of existing NCP materials (See Appendix 2 of attached IIR). We chose *not* to develop an attention control arm. Part of the process of the peer health coach intervention is the increased social support of the home visits. By providing "attention" in another way, we would not be able to test the effect of the increase in social support provided to intervention participants. Social support (or "attention") has been shown to be a powerful resource to maintain both physical and mental quality of life, despite the impacts of multiple chronic conditions.[67] Perceived social support has been significantly related to health-related quality of life.[67]

Follow-up calls for Intervention Group:

The same peer health coach will make a total of 10 phone calls over the 12 month intervention period (Table 3), including 5 phone calls (to replace the previous home visits) plus 5 telephone follow up calls. We are basing the intervention frequency and number of visits on our previous work (1R18DK088072-01) and on frequencies noted in successful blood pressure trials.[10] To increase generalizability of this model, we chose a minimal intervention necessary to review education topics and address important behaviors for all Veterans with hypertension. The peer health coach will support the participant in plan implementation at follow-up calls taking place 2, 4, 6 and 9 months after the initial call. Follow up telephone contacts are being used in our current study to maintain contact and support for participants.

Table 3. Schedule of home visits, telephone follow up and staff member responsible for the activity

Months	0	1	1.5	2	3	4	5	6	7	8	9	10	11	12	Staff member responsible for activity
**Baseline enrollment**	X														Research Assistant (RA)
**Telephone calls**		X		X		X		X			X				Peer health coach
**Telephone follow up**			X		X		X		X			X			Peer health coach
**Exit call**														X	RA,

Visit content and development of a health action plan:

All Vet-Coaches receive training in adherence to the study protocol and specific instruction on conducting research at the VA. Vet-Coaches complete the same online trainings in data security as well as the in-person Human Research Protection Program (HRPP) Orientation required by VA research staff. Vet-Coaches receive training in guidelines set by the VA and IRB to protect the rights of those participating in research, including an emphasis on maintaining participant confidentiality and keeping Personal Health Information secure at all times. Comprehensive trainings provide information on patient-centered communication, motivational interviewing, role modeling, comprehension of the health educational topics, health coaching skills, proper procedures for taking blood pressure measurements, goal setting techniques and proper

completion of data collection forms. Study staff will directly observe practice role play sessions prior to Vet-Coaches meeting with participants. Randomly selected encounters of Vet-Coach calls with participants will be audio recorded and reviewed by study staff to provide ongoing feedback to coaches. Individual performance feedback will be provided to ensure health coaching is being conducted correctly. In addition, study staff will conduct an annual, in person “Vet-Coach Refresher Training” with the Vet-Coaches that will review study protocol & limits of appropriate subject contact. The training will emphasize regulations and acceptable communications and contact with participants as set by both the VA Puget Sound employee code of conduct as well as the study protocol. There are bi-weekly meetings with the study team to discuss ongoing health coaching issues, problem-solve difficulties implementing study procedures or coordination of health care, and address concerns or questions specific to individual participants, etc.

Recommendations for potential health action plans, based on national guidelines and specific to the individual risk factor and patient motivation, will be generated at the enrollment visit. Using the results of the baseline assessment, the peer health coach will collaboratively develop an action plan with the Veteran. The protocols specify education content, Veteran skill development goals, participant and Veteran health coach actions. All Veterans will receive the five required education modules, including high blood pressure, medication adherence, physical activity, nutrition, and improved communication with medical team/physician (Table 2). The peer health coach will have participants prioritize their goals, ask what they would like to prioritize for their next call, and develop a goal-directed action plan [where the Veteran identifies a health related goal and decides how much time, when and how often they are going to complete the behavior (e.g. walk 10 minutes per day OR take my blood pressure pills every morning)], along with an assessment of how confident they feel in being able to complete the behavior. We will assess goal setting and goal completion rates in the Action Plan for the intervention participants. The peer health coach will document the skill or goal that was the primary focus for each call and the self-management strategies that were highlighted during that call on an encounter form. As progress toward the first goal is important for long-term motivation, a telephone follow up will occur 2 weeks after the first call. Blood pressure will be measured by the peer health coach at each call with a total of one blood pressure measurement at each call. The readings, if elevated, will be reviewed by the study coordinators and given to the provider. We will record these blood pressure readings in study data and use them to provide participant and PCP feedback. They will not be used as outcomes measures because we will not have equivalent data for the control participants. Initial and randomly selected phone calls with Vet-Coaches will be audio-recorded to ensure fidelity. Each Vet-Coach will audio-record initial calls until the PI deems they have met proficiency standards. After initial proficiency standards are met, each Vet-Coach will continue audio-recording of randomly-selected calls to ensure ongoing proficiency. Audio recordings will be collected using secure, VA approved digital recorders or recording software. Recordings and recording devices will be stored and transported in locked security bags to the VA Puget Sound office location every two weeks at study staff meetings. Audio recordings will then be uploaded to the secure, electronic folder on the VA server.

At follow up calls, the peer health coach will learn about patient challenges and concerns, review progress on implementing the self-management plan, provide targeted education to help with plan

implementation, revise self-management goals and the plan as the participant progresses, and make referrals to VHA and community resources. If deviations in medication adherence are noted, pillboxes will be offered. Blood pressure cuffs will be offered for home monitoring, as supported by a recent review of home blood pressure monitoring reporting moderate strength evidence.[68] Participants will receive assistance with health system navigation.

If an intervention participant moves out of the Puget Sound area and does not have an assigned VA provider, health coaching encounters will be discontinued because the PI would not be able to notify the provider of possible medical issues that may arise during the encounters. If a participant moves out of the area and retains a VA provider (at either the VA Puget Sound or a VA in another state), we would continue to complete health coaching encounters and the PI will report medical concerns to their assigned VA provider listed in CPRS. Study staff will continue to complete the exit phone visit with participants regardless of continuation of health coaching visits.

5.2 Recruitment Methods

- State how many subjects will be needed.
- Describe when, where, how and by whom potential subjects will be identified and recruited.
- Describe materials that will be used to recruit subjects, e.g., advertisements. Include materials as an appendix or separate attachment. Only refer to attachments as A, B, etc. Do not refer to version #'s or dates of versions. During the course of the study, changes in the attachments will require IRB approval; however, those changes may not require a revised protocol.
- Describe any payments to subjects, including the amount, timing (at the end of the study or pro-rated for partial study participation), method (e.g., cash, check, gift card), and whether subjects will experience a delay in receiving the payment.

Eligibility for participation will be determined by identification of potential participants and Vet-Coaches from VA administrative data files from VA Corporate Data Warehouse, as well as the "Quality Improvement for Primary Care Clinic" project conducted through the Seattle VA Primary Care Clinic for relevant diagnostic and lab-based criteria. Census tract data from the Quality Improvement project will be linked to subject geographic data (address) in the VA administrative files. This information will be obtained by the project programmer. Using administrative data, we identified approximately 17,080 Veterans at VA Puget Sound (cared for at either the Seattle or American Lake Divisions) with hypertension, of whom n=2,955 have a systolic blood pressure that is > 150 mm Hg. Approximately 90% of this population is overweight or obese (a second criteria for entry into the study) (n=2,486). Our target sample size for recruitment is n=400. A letter from the study signed by the primary care provider (PCP) with an opt-out option will be

mailed to potential participants informing them of the project and encouraging participation. Project staff (project manager and research assistant) will then contact patients by telephone to assess eligibility and interest in participation and to arrange an enrollment visit.

We will also recruit subjects by posting study flyers (see attached flyer with tabs) on research bulletin boards, research kiosks, and research information tables (on designated research fair days) at the VA Puget Sound. The flyer contains a contact number potential participants may call to learn more about the study from study staff. A copy of the study flyer without tabs will be included in the “Patient Education Programs” brochure, which is updated quarterly by the VA Health Behavior Coordinator and distributed at the VA Puget Sound. Vet-Coaches will also distribute copies of the study flyer and will be available to speak to interested potential participants in the PCC waiting room. All interested callers will be asked to complete the phone screen with study staff to determine eligibility. Use of the recruitment flyers will occur during business hours through December 31, 2020.

Informed consent will be obtained at the time of enrollment. To facilitate enrollment and retention, the project will offer $50 for completion of baseline data collection and $50 for completing one year exit data collection. We will not offer an incentive for participating in the intervention in order to maintain study generalizability and feasibility of wider adoption.

Vet-Coaches will be Veterans with VA employee or “WOC” status who have completed all the online VA Human Subjects trainings (including data security, privacy and confidentiality trainings) as well as in-depth instruction in health coaching and education. Vet-Coaches will serve as study staff to provide peer health coaching to Veteran subjects enrolled in the study. A letter will be sent to the provider of potential Vet-Coaches to identify candidates for this position (see attached letter to providers). The letter will include a list of eligible Vet-Coaches identified from the "Quality Improvement for Primary Care Clinic" project, and will ask providers to identify those on the list who would be good candidates to serve as Vet-Coaches. Selected candidates will be sent a recruitment letter to inquire if they would be interested in applying for a Vet-Coach position (see attached letter to potential Vet-Coaches). The project manager will conduct a follow up phone call to collect more information about their qualifications and interest in the position (see attached phone script), and the PI will conduct an in-person interview with the most qualified candidates to make a final hiring decision. Hired Vet-Coaches will be provided with an information statement at the end of the study to see if they would like to participate in a voluntary qualitative interview (see information statement approved in the initial IRB application and the following “patient and provider interviews” section in this protocol).

To facilitate enrollment and retention of eligible participants, the project will offer several incentives. After completion of the baseline visit, participants will receive an incentive of $50 and then $50 for completing year one follow-up data collection. Enrolled participants in the intervention group will receive a bag containing items that may encourage health related behavior including: a pill box/organizer; a scale; a place mat with information on healthy meal choices & portions (routinely distributed in the VA Primary Care Clinic); educational handouts (routinely distributed in the VA Primary Care Clinic); and a meal portion control tool.

See Attachment A (Recruitment Letter)

5.3 Informed Consent Procedures

- Indicate if informed consent will be obtained and/or if you are requesting a waiver of informed consent or waiver of documentation of informed consent. If the research involves multiple phases, specify for which phases of the research the waiver(s) is being requested and/or the informed consent will be sought.
- Describe who will be obtaining informed consent, if applicable, and any circumstances that may need to be addressed (e.g. subjects with impaired decision making ability and the use of a legally authorized representative, etc.)
- If applicable, indicate how local site study personnel will be trained regarding human subjects protections requirements and how to obtain and document informed consent.

Written informed consent will be obtained prior to enrollment by study staff (project manager and research assistant). At the enrollment visit, study staff will review a Commitment Script with potential participants prior to the consent process to discuss the study activities and commitments involved. This review will help participants determine whether the program is a good fit for them (in terms of the study activities, time commitments involved, etc.), and give them another chance to opt out prior to starting the consent process. The informed consent will also include information about audio-recording the Vet-Coach visits and phone calls as well as the semi-structured qualitative interviews. The consent will state that participants may decline to be audio-recorded at any time. Potential participants will be informed that participation is fully voluntary, that their decision regarding participation will in no way affect the services they receive, and that they may decline to answer any questions or decline any services offered as part of the project. Declining any one service will not affect their ability to receive other services from the project. They will also be informed that this study is a randomized trial and that they have an equal chance of receiving usual care or peer support intervention. The PI or study designee will review the study protocol and consent documents with all study personnel involved in consenting patients. Specifically, there will be an emphasis on explaining the enrollment process and clarifying that there are no consequences to their health care benefits whether they choose to participate or not.

5.4 Inclusion/Exclusion Criteria

- *Describe the criteria that determine who will be included in or excluded from the study.*

Participant Inclusion criteria: We will focus our intervention in the geographic area directly surrounding the VA Puget Sound (Seattle and ALVA clinics) that has a high prevalence of poorly controlled hypertension. As part of project planning, we have worked with the Office of Analytics and Business Intelligence to utilize Geographic Information Systems (GIS) to identify and target high risk areas. This type of patient mapping is available nationwide, so this mechanism to identify and recruit participants is potentially generalizable. Specific maps are shown in Appendix 1 of attached IIR.

We developed our inclusion criteria to identify Veterans who will benefit the most, by focusing on Veterans up to age 75 (no age restrictions below age 75) with poorly controlled risks that are amenable to change. Specific inclusion criteria include poorly controlled hypertension (mean systolic BP > 150/90 mmHg) and one or more of the following CVD risks: overweight or obese (Body Mass Index (BMI) $\geq$ 25 kg/m^2), current tobacco use, or a diagnosis of hyperlipidemia. Although we are selecting common conditions that increase cardiovascular risk, we are not excluding Veterans with other prevalent conditions (e.g. diabetes, arthritis, renal disease, or COPD), whose presence will be noted in the enrollment visit. We chose to focus on Veterans with elevations in systolic hypertension above the stage 1 level of 140 mmHg, given recent data regarding treatment of mild hypertension.

Exclusion criteria: We will exclude those who have been hospitalized 3 months prior to enrollment for a CVD admission (IHD, CVA or PVD), as these Veterans may be too ill to participate in this intervention. We will exclude patients with dementia or severe cognitive impairment that would not allow them to participate in the program; patients with a suicidal ideation and/or disruptive behavior flag in CPRS; patients with end stage renal disease on dialysis, given the unique care needs of this population; patients who have received palliative care or are enrolled in hospice care, given their limited life expectancy; Veterans enrolled in Home Based Primary Care, an all-inclusive home visit nurse program or those who live in care facilities (nursing homes, assisted living facilities); homeless Veterans and women who are pregnant or planning to become pregnant.

5.5 Study Evaluations

- Describe all evaluations to be conducted (including screening; tests/questionnaires that will be administered; any procedures that subjects will be required to complete) and data collection methods.

Include materials as an appendix or separate attachment. Only refer to attachments as A, B, etc. Do not refer to version #'s or dates of versions. During the course of the study, changes in the attachments will require IRB approval; however, those changes may not require a revised protocol.

The sources of data for this project will be participant interviews and medical records. The participant interviews will be collected specifically for this project while the medical record data will be obtained from existing sources. In order to evaluate the effectiveness of the proposed intervention, the study will administer baseline and exit interviews, conduct limited physical examinations where blood pressure, height and weight are measured, and, if needed, we will request the primary care provider order a blood test for potential participants. The proposed interview and limited physical exam will be administered at baseline and after the intervention period and will be conducted by study coordinators or research assistant. Only the study personnel will have access to any personally identifiable health information.

Measures

Specific Aim 1

Table 4 lists the health outcome measures. We will collect these outcome variables at baseline prior to randomization and at 12 months in both the intervention and control groups. Process and outcome data will be collected via the following sources: *Patient Surveys:* Baseline and 12-month surveys will be conducted by trained research staff. The administrative assistant and project manager will review all questionnaires and other data collection records, correct errors, and enter them into a password-protected database within 10 days of collection. *Semi-structured Interviews:* These will be conducted with purposive samples of Veterans upon exit from the program and at one year. Primary care staff will be interviewed after completing the intervention phase. *Time Logs:* Logs will be used to track time spent by the peer health coaches in intervention, attempting to reach patients, and key information about the content of those interactions. *Administrative and Clinical Data Systems:* These will be used to track patients' use of other VA inpatient and outpatient services.

Primary and secondary outcomes

The primary outcome measures will be systolic blood pressure. At the baseline visit and the final 12 month exit call, the study staff will ask the participant to measure blood pressure three times. At the time of scheduling, study staff will ask if the participant has a blood pressure monitor and/or scale in their possession. If the participant does not have a monitor or scale, staff will mail them one from the VA office. The mean value for these readings will be used for the baseline and exit measurements, respectively.

Secondary outcomes will include Framingham Risk Score (FRS), individual CVD risks (BMI, LDL-c, tobacco use), health care utilization and health-related quality of life. A 10-year risk of total CVD (including myocardial infarction, angina, coronary revascularization, stroke, congestive heart failure and peripheral arterial disease) will be determined according to FRS algorithms,[17] which include age, total and high-density lipoprotein-cholesterol, systolic blood pressure, treatment for hypertension, diabetes, and cigarette smoking. Individual components of this risk score are targets of the intervention (hypertension, hyperlipidemia, and smoking). The FRS algorithm has good predictive validity for CHD events (c statistics 0.74 and 0.77 for men

and women, respectively).[69] *Health* care utilization will include outpatient visits, emergency department visits, and hospitalizations. Health care utilization will be obtained from VA administrative data and by participant self-report to capture non-VA health care utilization. Health-related quality of life will be measured by the Medical Outcomes Study 12-item measure, the SF-12.[70] Two composite scales will be used to measure physical health (the physical component summary) and mental health (mental health component summary).[70]

Intermediate endpoints

a) Patient activation, self-efficacy and social support

An important intermediate endpoint is Veteran self-efficacy and patient activation. We hypothesize that exposure to **Vet-COACH** will improve chronic disease outcomes and lead to greater patient activation. We will use the Patient Activation Measure (PAM), a brief previously validated 13-item scale that assesses patient's self-rated ability to take preventive actions, manage symptoms of medical problems, find and use appropriate medical care, and work with their health care providers to make decisions about their care.[71] The PAM includes important constructs including self-efficacy and is not disease-specific. We will use the multi-dimensional scale of perceived social support to assess overall social support.[72,73] *In addition, we will utilize a measure of community support for healthy lifestyles (the Chronic Illness Resources Survey—Brief Form).[74]*

b) Self-management behaviors and patient-provider communication

Self-management behaviors will be measured with the following scales: The nutritional assessment of eating behavior will be a self-reported assessment "Starting the conversation", an eight-item validated, simplified food instrument designed for use in primary care and health-promotion settings.[60] We will utilize a food frequency questionnaire (FFQ) developed by the Nutrition Assessment Shared Resource of the Fred Hutchinson Cancer Research Center to measure nutritional intake. The FFQ provides estimates for 30 nutrients and has acceptable accuracy and reliability.[61] Intake of fruits and vegetables, fat, and fiber can be calculated based on published algorithms.[75] Level of physical activity will be assessed using the International Physical Activity Questionnaire (IPAQ), a validated and reliable measure of physical activity.[59] All participants will have an assessment of medication adherence using a standardized participant interview.[62] In addition, yearly medication adherence will be calculated from the electronic pharmacy data (ReComp).[63] Tobacco use will be assessed with standard questions from the Behavioral Risk Factor Surveillance Survey (BRFSS).[76] *We will assess alcohol consumption using the Alcohol Use Disorders Identification Test (AUDIT-C), a 3-item screening test for heavy drinking and/or abuse.[64]* We will ask about *utilization of stress management techniques[65] and* patient-provider communication using previously validated questionnaires (CAHPS).[66,77]

Table 4. Outcome Measures; measured at baseline and 12 months

	**Measurement domain**	**Measurement instrument**
**Primary outcome**		
Blood pressure	Systolic blood pressure (SBP)	Standard protocols[78]
**Secondary outcomes**		
Cardiovascular risk	Framingham risk score (FRS)	FRS algorithms[17]
Individual Cardio-metabolic risks	Body Mass Index (BMI)<br>Low density lipoprotein cholesterol (LDL-c)	Standard protocols for height and weight<br>Laboratory data from CPRS if within 3 months of enrollment, otherwise drawn by study RN at enrollment

Health Care Utilization	Outpatient and ER visits, hospitalizations, pharmacy utilization	Administrative data and participant self-report for non-VA care
Health-related quality of life	Role limitations caused by physical health, emotional problems, physical functioning, and general health	SF-12[70]
**Intermediate outcomes**		
Patient activation, Self-efficacy	Self-rated ability to take preventive actions, manage symptoms of medical problems, find and use appropriate medical care, and work with their health care providers	Patient Activation Measure (PAM)[71]
Social Support	Domains of Social support	Multidimensional Scale of Perceived Social Support[72]
Self-management Behaviors – Measure of behavioral enactment	Physical activity<br>Nutrition – eating behavior, food frequency<br>Medication adherence<br><br>Tobacco use<br>*Alcohol use*<br>*Stress Management*	IPAQ[59]<br>“Starting the conversation”[60],FFQ[61]<br>Voils, C. I. et al. Medication adherence questionnaire[62] ReCOMP[63] Medication Possession Ratio<br>BRFSS[76]<br>*AUDIT-C[64]*<br>*Utilization of stress management techniques[65]*
Patient provider communication	Communication with physician and health care team	Consumer Assessment of Health Plans (CAHPS)-patient-provider communication[66]

Specific Aim 3: Determine intervention costs

These costs will include all labor and materials (pamphlets, home BP monitors, etc.) used in the intervention. We will administer weekly time sheets for all study personnel to estimate the amount of time they spend in various activities, and attribute the cost of these activities to either intervention costs or trial-specific costs, which are excluded from the intervention cost.

Covariate data

We will collect data on potential covariates that have been shown in previous studies to be associated with self-care and quality of life. We will use these covariates in our analysis to describe the patient population and to adjust for any differences across randomized groups. Demographic covariates include age, gender, household income, marital status, education, primary language and race/ethnicity (American Indian or Alaskan Native, Asian, Pacific Islander, black, white and their ethnicity as Hispanic or non-Hispanic). We will assess health literacy using a brief validated 3 - item screener.[79] Depressive symptoms will be assessed using the PHQ-8.[80]

Measuring Intervention fidelity

Fidelity will be assessed to a) identify areas of needed improvement for ongoing remediation for the peer coaches through review of audio recordings (Section C.4.2); b) provide qualitative description of fidelity and needed changes to peer coaches at least quarterly (Section C.4.2); and c) include in the quantitative models to determine relationship between fidelity and outcomes (Section C.7.3). We will develop a manual for implementation of the intervention, very similar to our manual for our diabetes self-management trial (Appendix 3 of attached IIR). This manual consists of required and optional educational modules and educational materials that will be provided to the participant for each topic. *To assess if participants received the*

scheduled phone calls, the total number of phone calls will be recorded for each participant. Prior to study initiation and on a quarterly basis, peer support counselors must demonstrate competence in delivering content with fidelity (≥ 80% of components). We will require the peer health coaches to complete an encounter form for each intervention visit; which will consist of a checklist of required modules and if they were delivered. We have developed encounter forms for our current CHW study that will be adapted for the current study. The study coordinators will abstract participant records to assess whether each client received the interventions specified by protocols. Study staff (RA, study coordinators, or Co-I) will also directly review audio recordings of one call per quarter to assess intervention fidelity. Fidelity will be measured using an observation form, and feedback will be given to each Vet-Coach. Monthly control reports will ensure that each participant received the required components of the intervention.

Specific Aim 4: patient and provider interviews

We will use theoretical purposive sampling to identify three groups of Veterans for semi-structured interviews: 1) individuals who demonstrate significant improvement in hypertension control, 2) individuals without significant improvement and 3) individuals who discontinued the intervention after 1 or 2 sessions. Veterans within all groups will be interviewed upon exit (< 3 weeks of last attendance) and Veterans in groups 1 and 2 will also be interviewed one year post program completion. *We anticipate that interviewing 15 to 20 Veterans in each group (n = 45-60).* Groups will be purposively sampled to include both male and female Veterans. Sample interview guides are provided in Appendix 4 of attached IIR (Follow-up interviews questions will follow the same format and be developed from the initial interview findings). These participants will be contacted regarding willingness to participate in a 30-45 minute telephone based interview that will be audio recorded. The first 10–15 minutes will be open ended and elicit overall description of the participants' satisfaction with the intervention and experience with the program. Follow up questions and prompts will focus on specific aspects of the intervention and barriers and facilitators. Interviews will be conducted in the clinic either before or after scheduled medical visits or over the telephone, based on participant preference.

We will also interview a subset of PACT team members (including Vet-Coaches, PCPs, RN case managers, LPNs, and pharmacists) whose patients' were enrolled in the program. Because we want to determine potential barriers and facilitators to implementation from the provider *and PACT team perspective*, the interviews will focus on staff perceptions of how the intervention helped, how it could be improved, potential barriers they can identify in implementation, and what changes in implementation strategy could potentially improve uptake. Vet-Coaches and PACT team members will be asked to participate in voluntary qualitative interviews at the end of the study. They will be provided with an Information Statement detailing their subject rights and study activities they will be asked to participate in. Standard audio equipment will be used to record telephone-based interviews. At their exit interview, we will assess satisfaction with the program and offer opportunities to provide feedback. Although the exact number of subjects needed to generate meaningful qualitative results cannot be identified *a priori*, the main goals relate to developing deep case-oriented perspectives, selecting subjects with a range of characteristics and perspectives, and achieving saturation (meaning the point at which subsequent interviews fail to produce new findings) for overriding content themes. Even though the precise number of cases required to achieve thematic saturation cannot be predetermined, the clarity of the topic being studied, multiple interviews with participants over time, the use of focused research questions and researcher experience decrease the sample needed for meaningful findings. *We anticipate interviewing 18 - 24 providers and PACT team members will be sufficient for meaningful findings. We will use purposive sampling to include up*

to eight Vet-Coaches, 5 -7 PCPs, RN case managers, and LPNs, 3 staff Pharmacists, and 2 outpatient nutritionists.

Barriers and facilitators to implementation

We will collect information on the barriers and facilitators of implementation. We include features in the study implementation to promote external validity such as inclusive enrollment criteria and using the minimum intervention needed for change (e.g. implementing with the minimum intensity we believe necessary for effectiveness). We will ascertain the degree of participation by eligible Veterans. We will use the following measures: (1) ability to contact potential participants (# reached/ # contact attempts); (2) study enrollment rate (#enrolled in study/ #eligible reached); (3) study participation rate (# with at least one follow-up home visit or participation in one project group activity/ # eligible reached); (4) program dropout rate (# discharged from program/ # participating in program); (5) study completion rate (# completing exit data collection/ # enrolled in study). The project manager will track feasibility by logging program costs, FTE used, space used, and logistical barriers encountered. Program staff will complete time study logs weekly during the first six months of the project and then for one week every quarter. The program manager will collect data on adaptability by logging changes in the research model protocols as the program is implemented. We will assess components that worked well or did not through participant satisfaction questions at the exit interview, and semi-annual discussions among project staff and investigators.

5.6 Data Analysis

- Provide sample size determination and analysis (include anticipated rate of screen failures, study discontinuations, lost to follow-up etc.).
- Describe how, where and by whom the data will be analyzed.

Specific Aim 1

The primary outcomes are changes from baseline to one year in systolic blood pressure. We will test the primary hypothesis that among Veterans with poorly controlled hypertension and at least one other CVD risk, the **Vet-COACH** intervention targeting behavior change and disease self-management will decrease SBP by 4 mmHg compared to a usual care + education control group.

Baseline Analysis and descriptive statistics

Equivalence of participants in the intervention and control arms will be assessed on demographic and clinical variables, including outcomes, health status, co-morbidity, and utilization variables. Because this is a randomized controlled trial, no systematic bias is anticipated. However, we will conduct adjusted analyses and use as covariates those factors that are thought *a priori* to be related to the primary and secondary outcomes. We will report summary statistics for all baseline covariates and outcomes (means, standard deviations, and quartiles for continuous variables; frequencies and percentages for binary variables) by randomized group.

Primary Effectiveness Analyses for Specific Aims 1 & 2

Since there are multiple patients enrolled per physician and multiple patients enrolled per peer health coach, a physician-level and peer health coach random effect will be included in the

primary analytic model to take this correlation into account. For the Aim 1 primary outcome analysis, we will evaluate change in systolic blood pressure, from baseline to 12 months, in the intervention versus controls using the following random effects model:

$$Y_{ij} = \mu + \beta_1 Group_{ij} + \beta_2 Baseline_{ij} + \beta_3 x_{ij} + \alpha_j + Group_{ij} * \tau_k + \epsilon_{ijk}$$

where Y_{ij} *is the change in blood pressure from baseline to 12 months for subject i having provider j (and k for peer coach intervention participants);* μ *is the overall mean;* $Group_{ij}$ *is group assignment (intervention vs. control);* $Baseline_{ij}$ *is the baseline BP value;* x_{ij} *are baseline covariates that could affect the outcome as outlined in Section C.6.5;* α_j *is the random effect for provider cluster j and* τ_k *is the random effect for peer coach cluster k (note that* τ_k *is multiplied by the intervention indicator* $Group_{ij}$ *so that the added peer coach correlation is accounted for only amongst intervention participants, as suggest by Roberts et al*[81]*). Our main goal is to test whether* $\beta_1 = 0$ *which is the difference in BP change (from baseline to 12 months) between the intervention and control groups. All Aim 1 secondary outcomes and Aim 2 intermediate outcomes will be compared between intervention and control participants using the same model above for continuous outcomes and using a logit link for the same model for binary outcomes. We will include a sensitivity analysis that will explore whether intervention fidelity influences the effect of the intervention on primary outcomes, treatment receipt ("dose" of intervention: number of visits, phone calls, proportion of required modules delivered).*

Specific Aim 3

We will calculate intervention costs per patient. These costs will include all labor and materials (pamphlets, home BP monitors, etc.) used in the intervention divided by the number of participants.

Specific Aim 4 - Qualitative analysis

Transcripts will be analyzed using simultaneous deductive and inductive content analysis. Inductive content analysis consists of open/unstructured coding and allows for the identification of emergent previously unidentified or unexpected themes while deductive content analysis is more structured and consists of identifying meaning units that fit within pre-identified a-priori categories.[82] A-priori codes for patient interviews will include, but not be limited to: patient satisfaction, barriers and facilitators to participation, and acceptability. A-priori codes for *staff* interviews will include, but not be limited to: barriers and facilitators to implementation, and sustainability acceptability, and *changes in PACT team involvement in care*. Coding will be done while simultaneously listening to the audio file and reading the transcripts. Sub-codes will be developed by identifying broad themes followed by sub-coding schemes based on representative recordings. Quotes that do not accurately fit existing sub-codes will be used to develop new sub-codes iteratively. Qualitative data collection and analysis will be blind regarding participants' responder/non-responder status until the final merging stage of analysis. The results of the analysis process will be reviewed by members of the research team to assess their thoroughness and comprehensiveness for analytical rigor. Accountability will be supported by confirming results with respondents in subsequent interviews.

Missing data analyses

All appropriate efforts will be made to avoid missing data. Accruing data will be monitored for the presence of missing values, and the study coordinator will investigate causes and/or remedies for missing data. For key analysis variables that have 15% or more missing values, we will analyze patient and clinical factors that are associated with having a missing value. We will then perform analyses using multiple imputations to impute these key variables to allow

analysis for all subjects and reduce any bias associated with the missing data. We will perform a sensitivity analysis to determine if missing values have an effect on study outcomes.

Study power and sample size

We have powered our study to detect a clinically significant change in systolic blood pressure (SBP) of 5 mmHg) consistent with findings from other peer support and CHW studies.[83,84] The sample size we project is n= 354 Veterans, which assumes 80% power, 15% attrition, alpha 0.05, a PEER ICC 0.007, a provider ICC 0.015, provider cluster size of 5 (70 providers), PEER cluster size of 35 (5 PEER coaches).The SD of 14.2 mmHg was taken from a recently published review of CV risk factors among Veterans.[85] We estimate we will lose 15% of the sample (n-53), which would result in a final sample size of N=400(or 200in each arm), similar to our rate in our ongoing CHW trial[52] and previous CHW studies.[86,87] Because of the potential for multiple patients enrolled per physician, we have adjusted both sample size calculations for potential clustering by physicians within the clinic, using estimates of ICC = 0.015 based on other published hypertension trials among primary care practices[88,89] and clustering by Veteran peer health coach ICC = 0.007, based on a randomized trial of peer support among individuals with diabetes.[90] We will randomize n=200 Veterans to the control and n=200 Veterans to the intervention group (which will ultimately be reduced to 177 Veterans in the control and 177 Veterans in the intervention group after an estimated 15% loss of participants), to have a power of 80% to detect a difference of 5 mmHg in systolic blood pressure between groups.

5.7 Withdrawal of Subjects

- Describe any anticipated circumstances under which subjects will be withdrawn from the research without their consent.
- Describe the consequences of a subject's decision to withdraw from the research and the procedures for orderly termination of participation by the subject (e.g., the subject contacting the investigator for an end-of-study visit).

We do not anticipate any circumstances under which subjects will be withdrawn from the research without their consent. In our previous peer support trials, this has not occurred. The only circumstance where a participant would be withdrawn from the study would be in the event of threatening or unsafe behavior toward the study staff or peer health coaches. If a participant decides to stop the study for any reason, the intervention visits would end and study personnel would attempt to schedule an exit visit.

6.0 Reporting

`

- Include procedures for reporting unanticipated problems, serious adverse events, and protocol deviations.

Study progress and safety will be reviewed monthly (and more frequently if needed). An annual report will be compiled and will include a list and summarization of adverse events. In addition, the annual report will the study is justified on the basis that additional data are needed to accomplish the stated aims of the study; and (5) conditions whereby the study might be terminated prematurely. Safety information will be collected on the data collection forms at study visits, follow up phone calls and any additional contact with participants. PI will review all data collection (encounter) forms on an ongoing basis for data address (1) whether adverse event rates are consistent with pre-study assumptions; (2) reason for dropouts from the study; (3) whether all participants met entry criteria; (4) whether continuation of completeness and accuracy as well as protocol compliance. Encounter forms will be stored and transported in locked security bags immediately upon completion. These bags will be used to transport the study data to the VA Puget Sound office location every two weeks at study staff meetings. The encounter form will have a cover sheet containing the participant's name, address & phone number (for identification purposes), which will be detached from the pages of the form containing data upon receipt by study coordinators at the VA Puget Sound location. Encounter form pages containing study data will not contain any identifying information (such as name, phone number, etc.) Pages containing data have a study ID to link data. The cover sheet and study data will be stored separately in a locked cabinet. Safety data collection starts with enrollment of subjects. This includes study visits, follow up phone calls and any additional contact with participants.

The frequency of data review for this study differs according to the type of data and can be summarized as follows: Subject accrual (adherence to protocol regarding demographics, inclusion/exclusion) will be reviewed quarterly by the PI. Adverse event rates (injuries) will be reviewed quarterly by the PI. Compliance to treatment will be reviewed by study investigator and statistician bi-monthly.

7.0 Privacy and Confidentiality

- Describe whether the study will use or disclose subjects' Protected Health Information (PHI).
- Describe the steps that will be taken to secure the data (e.g., training, authorization of access, password protection, encryption, physical controls, Certificates of Confidentiality, and separation of identifiers and data)

Several steps will be taken to minimize the risk of invasion of privacy. Initial contact with prospective participants will be made via an introductory letter with a stamped post card allowing her/him to opt out of further contact by the study team thus limiting risk for invasion of privacy. A number of steps will be taken to ensure confidentiality and data protection throughout the rest of the study. All data gathered at the enrollment visit (the questionnaire, physical exam, health goals) and subsequent information will be confidential. Data will be kept in offices at VA Puget Sound HSR&D with locked filing cabinets and password protected computers. The investigators, project coordinator, and study staff will be the only staff to have access to confidential records. ID numbers rather than names will be used whenever possible. As part of the study, individuals will be reassured that information is to be kept confidential, no single individual will be identified by name and all data will be aggregated. All data files will be maintained on password protected PCs and password protected computer networks. Only aggregate data will be presented to external audiences. Individual identifiers will be deleted when they are no longer necessary for the project.

8.0 Communication Plan

- Include a plan for obtaining required coordinating site approval (if we are a participating site of a multi-site study).
- Include plan for keeping all engaged sites informed of changes to the protocol, informed consent, and HIPAA authorization, if we are the coordinating center. If we are a participating site, include a plan for how this information will be shared with you by the coordinating center.
- Include plan for informing local sites of any Serious Adverse Events, Unanticipated Problems, or interim results that may impact conduct of the study if we are the coordinating center. If we are a participating site, include a plan for how this information will be shared with you by the coordinating center.

N/A – We have 1 primary site.

9.0 Information Security and Privacy

The principal investigator will review critical incidents (participant death and hospitalization). For each such event, Dr. Nelson will evaluate the potential for causal relations between the incident and study or intervention procedures. Any adverse effects of the study will be reported to the participant and his/her health care provider. Major adverse events requiring hospitalization will be reported to

the study project manager and the IRB. Dr. Nelson will be available to meet with the participant and provider to discuss any concerns. The participants will bear no financial risk from any adverse effects of encounters from this study. Including only numeric identifiers on all data collection instruments will protect confidentiality of collected information. Code keys and data collection documents (when not in use) will be kept in a locked file or password-protected terminals.

Several steps will be taken to minimize the risk of invasion of privacy. Initial contact with prospective participants will be made via an introductory letter with a stamped post card allowing her/him to opt out of further contact by the study team thus limiting risk for invasion of privacy. A number of steps will be taken to ensure confidentiality and data protection throughout the rest of the study. All data gathered at the enrollment visit (the questionnaire, physical exam, health goals) and subsequent information will be confidential. Data will be kept in offices at VA Puget Sound HSR&D with locked filing cabinets and password protected computers. The investigators, project coordinator, and study staff will be the only staff to have access to confidential records. ID numbers rather than names will be used whenever possible. As part of the study, individuals will be reassured that information is to be kept confidential, no single individual will be identified by name and all data will be aggregated. All data files will be maintained on password protected PCs and password protected computer networks. Only aggregate data will be presented to external audiences. Individual identifiers will be deleted when they are no longer necessary for the project.

10.0 References

REFERENCES

1. Berry JD, Dyer A, Cai X, et al. Lifetime risks of cardiovascular disease. *N Engl J Med.* Jan 26 2012;366(4):321-329.

2. Vimalananda VG, Miller DR, Christiansen CL, Wang W, Tremblay P, Fincke BG. Cardiovascular disease risk factors among women veterans at VA medical facilities. *J Gen Intern Med.* Jul 2013;28 Suppl 2:S517-523.

3. Johnson ML, Pietz K, Battleman DS, Beyth RJ. Therapeutic goal attainment in patients with hypertension and dyslipidemia. *Medical care.* Jan 2006;44(1):39-46.

4. Lloyd-Jones DM, Hong Y, Labarthe D, et al. Defining and setting national goals for cardiovascular health promotion and disease reduction: the American Heart Association's strategic Impact Goal through 2020 and beyond. *Circulation.* Feb 2 2010;121(4):586-613.

5. Nelson KM. The burden of obesity among a national probability sample of veterans. *Journal of general internal medicine.* Sep 2006;21(9):915-919.

6. Brown DW. Smoking prevalence among US veterans. *J Gen Intern Med.* Feb 2010;25(2):147-149.

7. Viswanathan M, Kraschnewski JL, Nishikawa B, et al. Outcomes and costs of community health worker interventions: a systematic review. *Medical care.* Sep 2010;48(9):792-808.

8. Becker DM, Yanek LR, Johnson WR, Jr., et al. Impact of a community-based multiple risk factor intervention on cardiovascular risk in black families with a history of premature coronary disease. *Circulation.* Mar 15 2005;111(10):1298-1304.
9. Krieger J, Collier C, Song L, Martin D. Linking community-based blood pressure measurement to clinical care: a randomized controlled trial of outreach and tracking by community health workers. *American journal of public health.* Jun 1999;89(6):856-861.
10. Brownstein JN, Chowdhury FM, Norris SL, et al. Effectiveness of community health workers in the care of people with hypertension. *American journal of preventive medicine.* May 2007;32(5):435-447.
11. Morisky DE, Levine DM, Green LW, Shapiro S, Russell RP, Smith CR. Five-year blood pressure control and mortality following health education for hypertensive patients. *Am J Public Health.* Feb 1983;73(2):153-162.
12. Long JA, Jahnle EC, Richardson DM, Loewenstein G, Volpp KG. Peer mentoring and financial incentives to improve glucose control in African American veterans: a randomized trial. *Ann Intern Med.* Mar 20 2012;156(6):416-424.
13. Heisler M, Vijan S, Makki F, Piette JD. Diabetes control with reciprocal peer support versus nurse care management: a randomized trial. *Ann Intern Med.* Oct 19 2010;153(8):507-515.
14. Barber J, Rosenheck R, Armstrong M, Resnick S. Monitoring the Dissemination of Peer Support in the VA Healthcare System. *Community mental health journal.* 2008/12/01 2008;44(6):433-441.
15. Pfeiffer PN, Heisler M, Piette JD, Rogers MA, Valenstein M. Efficacy of peer support interventions for depression: a meta-analysis. *Gen Hosp Psychiatry.* Jan-Feb 2011;33(1):29-36.
16. Rosland AM, Nelson K, Sun H, et al. The patient-centered medical home in the Veterans Health Administration. *The American journal of managed care.* Jul 2013;19(7):e263-272.
17. D'Agostino RB, Sr., Vasan RS, Pencina MJ, et al. General cardiovascular risk profile for use in primary care: the Framingham Heart Study. *Circulation.* Feb 12 2008;117(6):743-753.
18. Schroeder SA. Shattuck Lecture. We can do better--improving the health of the American people. *N Engl J Med.* Sep 20 2007;357(12):1221-1228.
19. Danaei G, Ding EL, Mozaffarian D, et al. The preventable causes of death in the United States: comparative risk assessment of dietary, lifestyle, and metabolic risk factors. *PLoS medicine.* Apr 28 2009;6(4):e1000058.
20. Murray CJ, Lauer JA, Hutubessy RC, et al. Effectiveness and costs of interventions to lower systolic blood pressure and cholesterol: a global and regional analysis on reduction of cardiovascular-disease risk. *Lancet.* Mar 1 2003;361(9359):717-725.
21. Ford ES, Li C, Zhao G, Pearson WS, Capewell S. Trends in the prevalence of low risk factor burden for cardiovascular disease among United States adults. *Circulation.* Sep 29 2009;120(13):1181-1188.
22. Bambs C, Kip KE, Dinga A, Mulukutla SR, Aiyer AN, Reis SE. Low prevalence of "ideal cardiovascular health" in a community-based population: the heart strategies concentrating on risk evaluation (Heart SCORE) study. *Circulation.* Mar 1 2011;123(8):850-857.
23. Yoon J, Scott JY, Phibbs CS, Wagner TH. Recent trends in veterans affairs chronic condition spending. *Popul Health Manag.* Dec 2011;14(6):293-298.
24. van Berkel TF, Boersma H, Roos-Hesselink JW, Erdman RA, Simoons ML. Impact of smoking cessation and smoking interventions in patients with coronary heart disease. *European heart journal.* Dec 1999;20(24):1773-1782.
25. Hooper L, Summerbell CD, Thompson R, et al. Reduced or modified dietary fat for preventing cardiovascular disease. *Cochrane Database Syst Rev.* 2012;5:CD002137.

26. Chobanian AV, Bakris GL, Black HR, et al. The Seventh Report of the Joint National Committee on Prevention, Detection, Evaluation, and Treatment of High Blood Pressure: the JNC 7 report. *JAMA : the journal of the American Medical Association.* May 21 2003;289(19):2560-2572.
27. Blumenthal JA, Babyak MA, Hinderliter A, et al. Effects of the DASH diet alone and in combination with exercise and weight loss on blood pressure and cardiovascular biomarkers in men and women with high blood pressure: the ENCORE study. *Archives of internal medicine.* Jan 25 2010;170(2):126-135.
28. Whelton PK, Appel LJ, Espeland MA, et al. Sodium reduction and weight loss in the treatment of hypertension in older persons: a randomized controlled trial of nonpharmacologic interventions in the elderly (TONE). TONE Collaborative Research Group. *JAMA : the journal of the American Medical Association.* Mar 18 1998;279(11):839-846.
29. Effects of weight loss and sodium reduction intervention on blood pressure and hypertension incidence in overweight people with high-normal blood pressure. The Trials of Hypertension Prevention, phase II. The Trials of Hypertension Prevention Collaborative Research Group. *Archives of internal medicine.* Mar 24 1997;157(6):657-667.
30. Sacks FM, Svetkey LP, Vollmer WM, et al. Effects on blood pressure of reduced dietary sodium and the Dietary Approaches to Stop Hypertension (DASH) diet. DASH-Sodium Collaborative Research Group. *The New England journal of medicine.* Jan 4 2001;344(1):3-10.
31. Kelley GA, Kelley KS. Progressive resistance exercise and resting blood pressure : A meta-analysis of randomized controlled trials. *Hypertension.* Mar 2000;35(3):838-843.
32. Whelton SP, Chin A, Xin X, He J. Effect of aerobic exercise on blood pressure: a meta-analysis of randomized, controlled trials. *Ann Intern Med.* Apr 2 2002;136(7):493-503.
33. Xin X, He J, Frontini MG, Ogden LG, Motsamai OI, Whelton PK. Effects of alcohol reduction on blood pressure: a meta-analysis of randomized controlled trials. *Hypertension.* Nov 2001;38(5):1112-1117.
34. Bosworth HB, Olsen MK, McCant F, et al. Hypertension Intervention Nurse Telemedicine Study (HINTS): testing a multifactorial tailored behavioral/educational and a medication management intervention for blood pressure control. *Am Heart J.* Jun 2007;153(6):918-924.
35. Melnyk SD, Zullig LL, McCant F, et al. Telemedicine cardiovascular risk reduction in veterans. *Am Heart J.* Apr 2013;165(4):501-508.
36. McCall N, Cromwell J. Results of the Medicare Health Support disease-management pilot program. *N Engl J Med.* Nov 3 2011;365(18):1704-1712.
37. Crowley MJ, Powers BJ, Olsen MK, et al. The Cholesterol, Hypertension, And Glucose Education (CHANGE) study: Results from a randomized controlled trial in African Americans with diabetes. *Am Heart J.* Jul 2013;166(1):179-186 e172.
38. Heisler M, Hofer TP, Schmittdiel JA, et al. Improving blood pressure control through a clinical pharmacist outreach program in patients with diabetes mellitus in 2 high-performing health systems: the adherence and intensification of medications cluster randomized, controlled pragmatic trial. *Circulation.* Jun 12 2012;125(23):2863-2872.
39. Bosworth HB, Olsen MK, Dudley T, et al. Patient education and provider decision support to control blood pressure in primary care: a cluster randomized trial. *Am Heart J.* Mar 2009;157(3):450-456.
40. Wilson SR, Cram P. Another sobering result for home telehealth-and where we might go next. *Arch Intern Med.* May 28 2012;172(10):779-780.
41. Balcazar H, Lee Rosenthal E, Nell Brownstein J, Rush CH, Matos S, Hernandez L. Community Health Workers Can Be a Public Health Force for Change in the United

States: Three Actions for a New Paradigm. *American journal of public health.* 2011/12/01 2011;101(12):2199-2203.

42. Brownstein JN, Bone LR, Dennison CR, Hill MN, Kim MT, Levine DM. Community health workers as interventionists in the prevention and control of heart disease and stroke. *American journal of preventive medicine.* 2005;29(5):128-133.

43. Paul G, Smith SM, Whitford D, O'Kelly F, O'Dowd T. Development of a complex intervention to test the effectiveness of peer support in type 2 diabetes. *BMC Health Serv Res.* 2007;7:136.

44. Allen JK, Dennison Himmelfarb CR, Szanton SL, Frick KD. Cost-effectiveness of Nurse Practitioner/Community Health Worker Care to Reduce Cardiovascular Health Disparities. *J Cardiovasc Nurs.* Apr 30 2013.

45. Allicock M, Haynes-Maslow L, Carr C, Orr M, Kahwati LC, Weiner BJ. Training Veterans to Provide Peer Support in a Weight-Management Program: MOVE! *Preventing Chronic Disease.* 2013;10:E185.

46. Bandura A. Self-efficacy: Toward a unifying theory of behavioral change. *Psychological Review.* 1977;8:191-245.

47. Clark NM. Management of chronic disease by patients. *Annual review of public health.* 2003;24:289-313.

48. Leventhal L BY, Brownlee S, Diefenbach M, Leventhal EL, Patrick-Miller L, et al. *Illness representations: theoretical foundations. In Perceptions of health and illness.* : Harwood Academic Publisher.

49. Hibbard JH, Mahoney ER, Stock R, Tusler M. Do Increases in Patient Activation Result in Improved Self-Management Behaviors? *Health services research.* 2007;42(4):1443-1463.

50. Rollnick S, Mason P, Butler C. *Health Behavior Change*. Edinburgh: Churchill Livingstone; 2000.

51. van Dam HA, van der Horst FG, Knoops L, Ryckman RM, Crebolder HF, van den Borne BH. Social support in diabetes: a systematic review of controlled intervention studies. *Patient education and counseling.* Oct 2005;59(1):1-12.

52. Nelson K, Drain N, Robinson J, et al. Peer Support for Achieving Independence in Diabetes (Peer-AID): Design, methods and baseline characteristics of a randomized controlled trial of community health worker assisted diabetes self-management support. *Contemporary clinical trials.* Jul 2014;38(2):361-369.

53. Diao D, Wright JM, Cundiff DK, Gueyffier F. Pharmacotherapy for mild hypertension. *Cochrane Database Syst Rev.* 2012;8:CD006742.

54. Wing RR, Bolin P, Brancati FL, et al. Cardiovascular effects of intensive lifestyle intervention in type 2 diabetes. *The New England journal of medicine.* Jul 11 2013;369(2):145-154.

55. U.S. Department of Veterans Affairs, National Center for Health Promotion and Disease Prevention*, web-based educational materials.* [online]

56. Siebenhofer A, Jeitler K, Berghold A, et al. Long-term effects of weight-reducing diets in hypertensive patients. *Cochrane Database Syst Rev.* 2011(9):CD008274.

57. CDC, National Center for Health Statistics, *National Health and Nutrition Examination Survey 2011-2012, web-based.*[online]

58. Kim JW, Bosworth HB, Voils CI, et al. How well do clinic-based blood pressure measurements agree with the mercury standard? *J Gen Intern Med.* Jul 2005;20(7):647-649.

59. Hagstromer M, Oja P, Sjostrom M. The International Physical Activity Questionnaire (IPAQ): a study of concurrent and construct validity. *Public health nutrition.* Sep 2006;9(6):755-762.

60. Paxton AE, Strycker LA, Toobert DJ, Ammerman AS, Glasgow RE. Starting the conversation performance of a brief dietary assessment and intervention tool for health professionals. *American journal of preventive medicine.* Jan 2011;40(1):67-71.

61. Thompson FE, Midthune D, Subar AF, Kahle LL, Schatzkin A, Kipnis V. Performance of a short tool to assess dietary intakes of fruits and vegetables, percentage energy from fat and fibre. *Public health nutrition.* Dec 2004;7(8):1097-1105.

62. Voils, C. I., Maciejewski, M. L., Hoyle, R. H., Reeve, B. B., Gallagher, M. P., Bryson, C. L., & Yancy Jr., W. S. (2012). Initial validation of a self-report measure of the extent of and reasons for medication nonadherence. Medical Care, 50(12), 1013-1019. PMID 22922431.**63.** Bryson CL, Au DH, Young B, McDonell MB, Fihn SD. A refill adherence algorithm for multiple short intervals to estimate refill compliance (ReComp). *Medical care.* Jun 2007;45(6):497-504.

64. Bush K, Kivlahan DR, McDonell MB, Fihn SD, Bradley KA. The AUDIT alcohol consumption questions (AUDIT-C): an effective brief screening test for problem drinking. *Archives of internal medicine.* 1998;158:1789-1795.

65. Lorig K SA, Ritter P, González V, Laurent D, Lynch J. *Outcome Measures for Health Education and other Health Care Interventions.* Thousand Oaks CA: Sage Publications; 1996.

66. Solomon LS, Hays RD, Zaslavsky AM, Ding L, Cleary PD. Psychometric properties of a group-level Consumer Assessment of Health Plans Study (CAHPS) instrument. *Medical care.* Jan 2005;43(1):53-60.

67. Fortin M, Bravo G, Hudon C, et al. Relationship between multimorbidity and health-related quality of life of patients in primary care. *Quality of life research : an international journal of quality of life aspects of treatment, care and rehabilitation.* Feb 2006;15(1):83-91.

68. Uhlig K, Patel K, Ip S, Kitsios GD, Balk EM. Self-Measured Blood Pressure Monitoring in the Management of Hypertension: A Systematic Review and Meta-analysis. *Ann Intern Med.* Aug 6 2013;159(3):185-194.

69. Wilson PW, D'Agostino RB, Levy D, Belanger AM, Silbershatz H, Kannel WB. Prediction of coronary heart disease using risk factor categories. *Circulation.* May 12 1998;97(18):1837-1847.

70. Ware J, Snow K, Kosinski M, Gandek B. *SF-36 Health Survey. Manual and Interpretation Guide.*: The Health Institute, New England Medical Center, Boston, MA; 1993.

71. Hibbard JH, Stockard J, Mahoney ER, Tusler M. Development of the Patient Activation Measure (PAM): conceptualizing and measuring activation in patients and consumers. *Health services research.* Aug 2004;39(4 Pt 1):1005-1026.

72. Zimet GD, Powell SS, Farley GK, Werkman S, Berkoff KA. Psychometric characteristics of the Multidimensional Scale of Perceived Social Support. *Journal of personality assessment.* Winter 1990;55(3-4):610-617.

73. Vaglio J, Jr., Conard M, Poston WS, et al. Testing the performance of the ENRICHD Social Support Instrument in cardiac patients. *Health Qual Life Outcomes.* May 13 2004;2:24.

74. Glasgow RE, Strycker LA, Toobert DJ, Eakin E. A social-ecologic approach to assessing support for disease self-management: the Chronic Illness Resources Survey. *J Behav Med.* Dec 2000;23(6):559-583.

75. National Cancer Institute, Applied Research, Cancer Control and Population Sciences, *web-based Dietary Screener in 2010 NHIS Cancer Control Supplement.* [online]

76. Centers for Disease Control and Prevention. *Web-based Overview: BRFSS 2000*. [online]

77. Fischer D, Stewart AL, Bloch DA, Lorig K, Laurent D, Holman H. Capturing the patient's view of change as a clinical outcome measure. *JAMA : the journal of the American Medical Association.* Sep 22-29 1999;282(12):1157-1162.

78. Pickering TG, Hall JE, Appel LJ, et al. Recommendations for blood pressure measurement in humans and experimental animals: Part 1: blood pressure measurement in humans: a statement for professionals from the Subcommittee of Professional and Public Education of the American Heart Association Council on High Blood Pressure Research. *Hypertension.* Jan 2005;45(1):142-161.

79. Chew LD, Griffin JM, Partin MR, et al. Validation of screening questions for limited health literacy in a large VA outpatient population. *Journal of general internal medicine.* May 2008;23(5):561-566.

80. Kroenke K, Strine TW, Spitzer RL, Williams JB, Berry JT, Mokdad AH. The PHQ-8 as a measure of current depression in the general population. *J Affect Disord.* Apr 2009;114(1-3):163-173.

81. Roberts C, Roberts SA. Design and analysis of clinical trials with clustering effects due to treatment. *Clin Trials.* 2005;2(2):152-162.

82. Elo S, Kyngäs H. The qualitative content analysis process. *Journal of Advanced Nursing.* 2008;62(1):107-115.

83. Krantz MJ, Coronel SM, Whitley EM, Dale R, Yost J, Estacio RO. Effectiveness of a community health worker cardiovascular risk reduction program in public health and health care settings. *American journal of public health.* Jan 2013;103(1):e19-27.

84. Levine DM, Bone LR, Hill MN, et al. The effectiveness of a community/academic health center partnership in decreasing the level of blood pressure in an urban African-American population. *Ethnicity & disease.* Summer 2003;13(3):354-361

85. Goldstein KM, Melnyk SD, Zullig LL, et al. Heart matters: Gender and racial differences cardiovascular disease risk factor control among veterans. *Women's health issues : official publication of the Jacobs Institute of Women's Health.* Sep-Oct 2014;24(5):477-483

86. Brown SA, Garcia AA, Kouzekanani K, Hanis CL. Culturally competent diabetes self-management education for Mexican Americans: the Starr County border health initiative. *Diabetes care.* Feb 2002;25(2):259-268.

87. Gary TL, Bone LR, Hill MN, et al. Randomized controlled trial of the effects of nurse case manager and community health worker interventions on risk factors for diabetes-related complications in urban African Americans. *Preventive medicine.* Jul 2003;37(1):23-32.

88. Bosworth HB, Olsen MK, Neary A, et al. Take Control of Your Blood Pressure (TCYB) study: a multifactorial tailored behavioral and educational intervention for achieving blood pressure control. *Patient education and counseling.* Mar 2008;70(3):338-347.

89. Powers BJ, King JL, Ali R, et al. The Cholesterol, Hypertension, and Glucose Education (CHANGE) study for African Americans with diabetes: study design and methodology. *Am Heart J.* Sep 2009;158(3):342-348.

90. Smith SM, Paul G, Kelly A, Whitford DL, O'Shea E, O'Dowd T. Peer support for patients with type 2 diabetes: cluster randomised controlled trial. *BMJ.* 2011;342:d715.
