# Supplementary material for: Effect of a Peer Health Coaching Intervention on Clinical Outcomes Among US Veterans With Cardiovascular Risks: The Vet-COACH Randomized Clinical Trial
Source: JAMA Netw Open. 2023 Jun 6;6(6):e2317046. doi: 10.1001/jamanetworkopen.2023.17046 (PMC10245194; doi:10.1001/jamanetworkopen.2023.17046)
Supplement: Supplement 2. — eMethods. Multiple Imputation of MCS and PCS eTable 1. Baseline Characteristics of Trial Participants by Missing SF-12 Status eTable 2. Change in MCS/PCS Between VetCOACH Intervention and Control Groups Using Completers Vs. Multiple Imputation eTable 3. Distribution of Completion of Core Visits Across Study Participants eTable 4. Pre-/Post-Covid Differences in Primary and Secondary Outcomes Between VetCOACH Intervention and Control Groups eTable 5. Pre-Covid Health Care Use Outcomes eTable 6. Post-Covid Health Care Use Outcomes [file jamanetwopen-e2317046-s002.pdf]

Supplemental Online Content

Nelson KM, Taylor L, Williams JL, et al. Effect of a peer health coaching intervention on clinical outcomes among US veterans with cardiovascular risks: the Vet-COACH randomized clinical trial. *JAMA Netw Open*. 2023;6(6):e2317046. doi:10.1001/jamanetworkopen.2023.17046

eMethods. Multiple Imputation of MCS and PCS

eTable 1. Baseline Characteristics of Trial Participants by Missing SF-12 Status

eTable 2. Change in MCS/PCS Between VetCOACH Intervention and Control Groups Using Completers Vs. Multiple Imputation

eTable 3. Distribution of Completion of Core Visits Across Study Participants

eTable 4. Pre-/Post-Covid Differences in Primary and Secondary Outcomes Between VetCOACH Intervention and Control Groups

eTable 5. Pre-Covid Health Care Use Outcomes

eTable 6. Post-Covid Health Care Use Outcomes

This supplemental material has been provided by the authors to give readers additional information about their work.

© 2023 Nelson KM et al. *JAMA Network Open*.

eMethods. Multiple Imputation of MCS and PCS

For key analysis variables that had 15% or more missing values, we analyzed demographic and clinical factors associated with having a missing value (eTable 1 below). We then performed analyses using multiple imputations to impute these key variables to allow analysis for all subjects and reduce any bias associated with the missing data.

SF-12 physical component summary (PCS) and mental component summary (MCS) values at 12 months had 16% missing (being the only variables to reach this threshold for missing in our data). We therefore used multiple imputation via chained equations using the mice package in R, conditioning on provider, coach, baseline MCS and PCS, final MCS (or final PCS), baseline demographics (age, sex, race, education, marital status, employment, income), and baseline clinical characteristics (cholesterol, HDL, diastolic blood pressure, systolic blood pressure, and body mass index). Diagnostics checks for plausible imputations and convergence were inspected to ensure quality of the imputations. N=47 Veterans were missing either baseline or 12-month SF-12 and therefore N=217 observations were used in the complete-data analyses.

© 2023 Nelson KM et al. *JAMA Network Open*

**eTable 1.** Baseline Characteristics of Trial Participants by Missing SF-12 Status		
	**Observed SF-12 (N=217)**	**Missing SF-12 (N=47)**
Demographic characteristics		
Assigned Intervention, n (%)	106 (49%)	28 (60%)
Male, n (%)	185 (82%)	44 (94%)
Age (years), mean (SD)	61 (9)	59 (11)
High school education or less, n (%)	39 (18%)	8 (18%)
Married, n (%)	88 (41%)	16 (36%)
Employed, n (%)	85 (40%)	15 (33%)
Retired, n (%)	80 (37%)	17 (38%)
Unable to work, n (%)	29 (13%)	8 (18%)
Annual income<=$40,000, n (%)	97 (49%)	20 (49%)
Hispanic, n (%)	17 (8%)	0 (0%)
Race, n (%)		
White, non-Hispanic	114 (81%)	24 (55%)
Black, non-Hispanic	66 (35%)	16 (36%)
Multiracial	31 (14%)	3 (6%)
Clinical characteristics		
Systolic BP, mean (SD)	137 (18)	133 (16)
Diastolic BP, mean (SD)	81 (10)	81 (11)
BMI, mean (SD)	32 (6)	31 (7)
Cholesterol, mean (SD)	180 (48)	182 (47)
HDL, mean (SD)	49 (16)	49 (16)
*Notes: BP = Blood Pressure; HDL = High-Density Lipoprotein; BMI = Body Mass Index		

The non-imputed “completers” analysis for MCS (n=47 missing) shows a statistically significant treatment effect (randomized group coefficient of 3.40; 95% CI: (0.98, 5.83); p=0.007), similar to the multiple imputation results for MCS reported in the manuscript (randomized group coefficient of 3.64, 95%CI: (0.66,6.63), p=0.02); the non-imputed “completers” analysis for PCS (n=47 missing) shows a non-significant treatment effect (randomized group coefficient of 0.30; 95% CI: (-1.84,2.46); p=0.79), similar to the multiple imputation results for PCS reported in the manuscript (randomized group coefficient of 0.51, 95%CI: (-1.76,2.77), p=0.67).

© 2023 Nelson KM et al. *JAMA Network Open*

**eTable 2.** Change in MCS/PCS between VetCOACH intervention and control groups using Completers Vs. Multiple Imputation		
	Adjusted Difference in Differences (95% CI), p-value	
	Completers (N=47 missing; N=217 observed)	Multiple Imputation (N=264)
Change in MCS	3.40 (0.98,5.83), p=0.007	3.64 (0.66,6.63), p=0.02
Change in PCS	0.30 (-1.84,2.46), p=0.79	0.51 (-1.76,2.77), p=0.66

© 2023 Nelson KM et al. *JAMA Network Open*

eTable 3. Distribution of completion of core visits across study participants

Visit #	Frequency	Percent	Cumulative Frequency	Cumulative Percent
**0**	13	9.70	13	9.70
**1**	9	6.72	22	16.42
**2**	5	3.73	27	20.15
**3**	5	3.73	32	23.88
**4**	6	4.48	38	28.36
**5**	2	1.49	40	29.85
**6**	9	6.72	49	36.57
**7**	8	5.97	57	42.54
**8**	4	2.99	61	45.52
**9**	15	11.19	76	56.72
**10**	58	43.28	134	100.00

© 2023 Nelson KM et al. *JAMA Network Open*

**eTable 4.** Pre-/post-covid differences in primary and secondary outcomes between VetCOACH intervention and control groups				
	Intervention Effect PRE-COVID		Intervention effect POST-COVID	
Change Measure	*Adjusted Difference (95% CI)*	*P value*	*Adjusted Difference in Differences (95% CI)*	*P value*
Systolic BP, Mean (SD)	-3.58 (-11.01,3.99)	0.36	-0.11 (-6.12,5.87)	0.97
Diastolic BP, Mean (SD)	-2.22 (-6.45,1.69)	0.30	-0.02 (-4.36,4.34)	0.99
% <=120/80, n (%)	OR (95% CI) 2.22 (0.78,6.27)	0.13	OR (95%CI) 0.59 (0.22, 1.58)	0.29
Change in Framingham, Mean(SD)	-0.01 (-0.05,0.03)	0.54	-0.0005 (-0.03,0.03)	0.98
Change in BMI, Mean (SD)	0.01 (-1.23,1.29)	0.99	-0.51 (-1.82,0.64)	0.42
LDL cholesterol, Mean (SD)	1.06 (-9.46,11.80)	0.84	-5.24 (-15.56,4.08)	0.34
Smoker, n (%)	Failed to converge*	--	OR (95% CI) = 0.72 (0.14,3.6)	0.70
Change in HRQOL MCS, Mean (SD); multiple imputation used	2.49 (-9.48,14.47)	0.69	3.62 (0.45,6.78)	0.03
Change in HRQOL PCS, Mean (SD); multiple imputation used	0.86 (-7.27,9.00)	0.84	-1.25 (-4.52,2.02)	0.46

* Too few events

**eTable 5.** Pre-Covid Health Care Use Outcomes					
	Control (n=66)		Intervention (n=70)		*Adjusted Difference in Differences (95% CI)*
Measure	*Baseline*	*12-month*	*Baseline*	*12-month*	
Outpatient visits/past year, mean (SD)	0.38 (0.78)	0.59 (1.62)	0.49 (1.26)	0.37 (0.94)	-0.141 (-0.579,0.185)
ED visits/past year, mean (SD)	1.27 (1.78)	0.83 (1.60)	0.99 (1.73)	0.89 (1.39)	0.121 (-0.366,0.602)
Hospitalized/past year, mean (SD)	0.09 (0.29)	0.09 (0.29)	0.20 (0.53)	0.11 (0.40)	Failed to Converge*

* Too few events

© 2023 Nelson KM et al. *JAMA Network Open*

**eTable 6.** Post-Covid Health Care Use Outcomes					
	Control (n=64)		Intervention (n=64)		*Adjusted Difference in Differences (95% CI)*
Measure	*Baseline*	*12-month*	*Baseline*	*12-month*	
Outpatient visits/past year, mean (SD)	0.70 (1.62)	0.50 (1.02)	0.44 (1.02)	0.42 (0.85)	-0.016 (-0.369,0.231)
ED visits/past year, mean (SD)	1.14 (2.56)	0.89 (1.72)	1.08 (2.23)	0.83 (1.35)	-0.052 (-0.515,0.497)
Hospitalized/past year, mean (SD)	0.11 (0.44)	0.17 (0.61)	0.20 (0.51)	0.20 (0.65)	Failed to Converge*

* Too few events

© 2023 Nelson KM et al. *JAMA Network Open*
