# Supplementary material for: Effect of a Peer Health Coaching Intervention on Clinical Outcomes Among US Veterans With Cardiovascular Risks: The Vet-COACH Randomized Clinical Trial
Source: JAMA Netw Open. 2023 Jun 6;6(6):e2317046. doi: 10.1001/jamanetworkopen.2023.17046 (PMC10245194; doi:10.1001/jamanetworkopen.2023.17046)
Supplement: Supplement 3. — Data Sharing Statement [file jamanetwopen-e2317046-s003.pdf]

Data Sharing Statement

Nelson. Effect of a Peer Health Coaching Intervention on Clinical Outcomes Among US Veterans With Cardiovascular Risks. *JAMA Netw Open*. Published June 06, 2023. doi:10.1001/jamanetworkopen.2023.17046

Data
Data available: No

Additional Information
Explanation for why data not available: VHA data
